# Identification of genes associated with hepatitis B virus infection and breast cancer tumorigenesis and progression

**DOI:** 10.1016/j.bbrep.2025.102156

**Published:** 2025-07-14

**Authors:** Meirong Zhou, Lili Xu, Haonan Jiang, Lejia Xiong, Shuangping Zhou, Danhua Zhang, Jia Yao, Mei Dai, Lun Li

**Affiliations:** a Department of General Surgery, The Second Xiangya Hospital, Central South University, Changsha, China; b Clinical Research Center for Breast Disease in Hunan Province, Changsha, China; c Xiangya School of Medicine, Central South University, Changsha, China

**Keywords:** Hepatitis B virus, Breast cancer, Meta analysis

## Abstract

**Background:**

Studies found that Hepatitis B virus (HBV) infection might be a risk factor for breast cancer tumorigenesis and progression, but the potential mechanism of HBV in breast cancer tumorigenesis and progression was unclear. Our study aimed to understand the interplay between HBV infections and breast cancer via RNA seq data analysis.

**Methods:**

We searched GEO databases for related databases. Those datasets that analyzed RNAseq data between breast cancer tissues and normal breast tissues or with prognostic information were included. In vivo cell experiments were conducted to validate the effect of HBV infection on breast cancer cells.

**Results:**

We retrieved 20 databases (2206 breast cancer tissues and 860 normal breast tissues) for differentiated expressed genes (DEGs) between breast cancer tissues and normal breast tissues. 54 datasets for survival analysis (14,518 breast cancer patients) were obtained. Higher expression of seven genes (BIRC5, CASP3, CCNA2, CCNE1, CXCL8, CYCS, E2F1) were associated with worse survival. HBV infection may lead to increased expression levels of several key genes in breast cancer cell lines, including CDK2, PCNA, CCNE2, CXCL8, E2F1, and CASP3.

**Conclusion:**

HBV might lead to the changes of some genes in breast tissues, which might participate in breast cancer tumorigenesis and progression.

## Highlights

• Some of HBV-related genes exhibit differences between breast cancer tissues and normal tissues.
• HBV-related genes may be linked to survival outcomes in breast cancer patients.
• HBV-related genes may play a role in the tumorigenesis and progression of breast cancer.

## 1 Background

Breast cancer is the most commonly diagnosed cancer among women worldwide. According to the World Health Organization, approximately 2.3 million women were diagnosed with breast cancer in 2020, making up 11.7 % of all new cancer cases [1]. Several factors affect the risk of developing breast cancer, including demographic, genetic, hormonal, and lifestyle factors. Some viruses have been associated with various types of cancer, but it remains unclear how these viruses are connected to breast cancer. Hepatitis B virus (HBV) infection remains a significant global health concern due to its potential to cause chronic liver disease, cirrhosis and liver cancer [2]. Approximately 254 million people worldwide are chronically infected with HBV [3]. Li et al. discovered that the prevalence of HBV infection was higher in the breast cancer group than in both the benign breast disease group and the healthy female group [4]. HBV antigens, viral DNA and RNA, and specific viral particles were found in breast cancer tissues [5]. Viruses can contribute to cancer through several mechanisms, including disrupting genes (insertional mutagenesis), causing chronic inflammation, and evading the immune system. HBV-DNA integration into the host genome has been shown to occur early in the clonal expansion of tumors. This process can lead to genomic instability and cause direct insertional mutagenesis in various cancer-related genes.

While hepatitis B virus (HBV) infection and breast cancer are distinct health issues, ongoing research is investigating their possible link, particularly regarding how HBV may affect tumor development and cancer progression [[6], [7], [8]]. HBsAg-positive breast cancer patients showed significantly worse disease-free survival (DFS) and overall survival (OS) compared to their HBsAg-negative counterparts [9]. Currently, there are no studies that clarify how the hepatitis B virus (HBV) impacts breast cancer biology and patient survival. Understanding the relationship between viral infections and breast cancer could lead to better management strategies and improved outcomes for patients facing both conditions. In this study, we aimed to analyze the HBV-related genes involved in breast cancer tumorigenesis and progression.

## 2 Methods

### 2.1 Gene sources

Hepatitis B virus related genes were retrieved from KEGG [10]. All genes in hsa05161 (Hepatitis B) were analyzed.

### 2.2 Data sources

We searched GEO databases using “breast cancers” OR “breast cancer” OR “breast neoplasm” OR “breast neoplasms” OR “breast tumor” OR “breast tumors” OR “breast adenocarcinoma”. Datasets that analyzed RNA seq data between breast cancer tissues and normal breast tissues were included. Finally we retrieved 20 databases. These were 2206 breast cancer tissues and 860 normal breast tissues (Table S1).

We obtained 54 datasets for survival analysis. There were 14,518 breast cancer patients (2862 overall survival (OS) events and 828 disease free survival (DFS) events, 1074 distant metastasis free survival (DMFS) events, 2088 recurrence free survival (RFS) events) (Table S2).

### 2.3 Data analysis

We calculated average (mean) and variance (standard deviation) values for breast cancer and normal breast tissues for each gene in different databases. The mean differences (MD) and 95 % confidence intervals (95 %CI) were analyzed based on 20 databases. The hazards ratios (HR) and 95 % confidence intervals (95 %CI) of OS, DFS, DMFS, RFS for each gene. Meanwhile, the prognostic values of these genes were validated by bc-GenExMiner v5.1 [11] and KMplot [12]. All data was pooled using random effect meta analysis methods by R software. P < 0.05 was considered statistically significant.

### 2.4 GO term and KEGG pathway enrichment analysis

The deferentially expressed genes (DEGs) were subjected to functional enrichment analysis in the Database for Annotation, Visualization and Integrated Discovery (DAVID) [13]. Through GO term enrichment and KEGG pathway enrichment analysis, we explored the biological significance of DEGs based on biological processes and KEGG pathway analysis. A P value < 0.05 was set as the threshold for significance. Enrichr was also used to predict candidate drug for targeting oncogene for breast cancer associated with worse survival [14].

### 2.5 RNAseq analysis

The study collected breast cancer and adjacent non-tumorous tissue samples from six triple negative breast cancer (TNBC) patients, with three patients infected with HBV and three without. For RNA sequencing, three breast cancer tissues and three adjacent non-tumorous tissues were obtained from HBV-infected patients. From non-HBV-infected patients, three breast cancer tissues and only two adjacent non-tumorous tissues (due to a higher fat content in the samples) were included in the analysis.

### 2.6 In vivo cell experiment

In order to validate the effect of HBV infection on breast cancer cells, we conducted in vivo cell experiments using HepG2.2.15 cell lines. HepG2.2.15 is a human liver cancer cell line that was developed by transfecting HepG cells with a recombinant plasmid containing two connected ends of full-length HBV-DNA. This cell line is capable of asexual proliferation in vitro and continuously and stably secretes HBsAg, HBeAg, and complete Dane particles in the culture medium. Additionally, HepG2.2.15 produces HBV mRNA of various lengths, including 3.5 kb, 2.5 kb, and 2.1 kb. For the experiment, HepG2.2.15 cells were cultured in Minimum Essential Medium (MEM) supplemented with 10 % fetal bovine serum (FBS), 1 % penicillin-streptomycin (P/S), and 0.4 mg/mL G418. The supernatant from HepG2.2.15 was then used to treat different breast cancer cell lines (BT474, SKBR3, MDA-MB-231, and MCF7). After 48 h, we collected the cells, extracted RNA, and analyzed changes in molecular expression. The molecules assessed included BAX, BIRC5, CASP3, CCNA2, CCNE1, CCNE2, CDK2, CXCL8, E2F1, E2F2, E2F3, and PCNA, as well as SRC. RNA extraction was performed using the NcmSpin Cell/Tissue Total RNA Kit (NCM Biotech, M5105), followed by reverse transcription using the Hifair® Ⅲ 1st Strand cDNA Synthesis SuperMix for qPCR (gDNA digester plus) from Yeasen Biotechnology (Shanghai) Co., Ltd. (11141 ES). This methodology allows for a comprehensive examination of how HBV influences gene expression in breast cancer cells, which could provide insight into the molecular mechanisms underlying the interplay between HBV infection and breast cancer progression.

For proliferation assay, cells were seeded in 96-well flat-bottomed plates with each well containing 1000 cells in 200 μl of culture medium. Cell viability was measured using the Cell Counting Kit 8 (C6005, NCM Biotech) according to manufacturer's instructions. Plates were read at 450 nM absorbance. For the clonal formation assay, 800 cells were seeded in 6 well plates. Plates were stained with 1 % crystal violet two weeks after seeding.

## 3 Results

### 3.1 Hepatitis B virus-associated genes between breast cancer and normal tissues

**Fig. 1:**
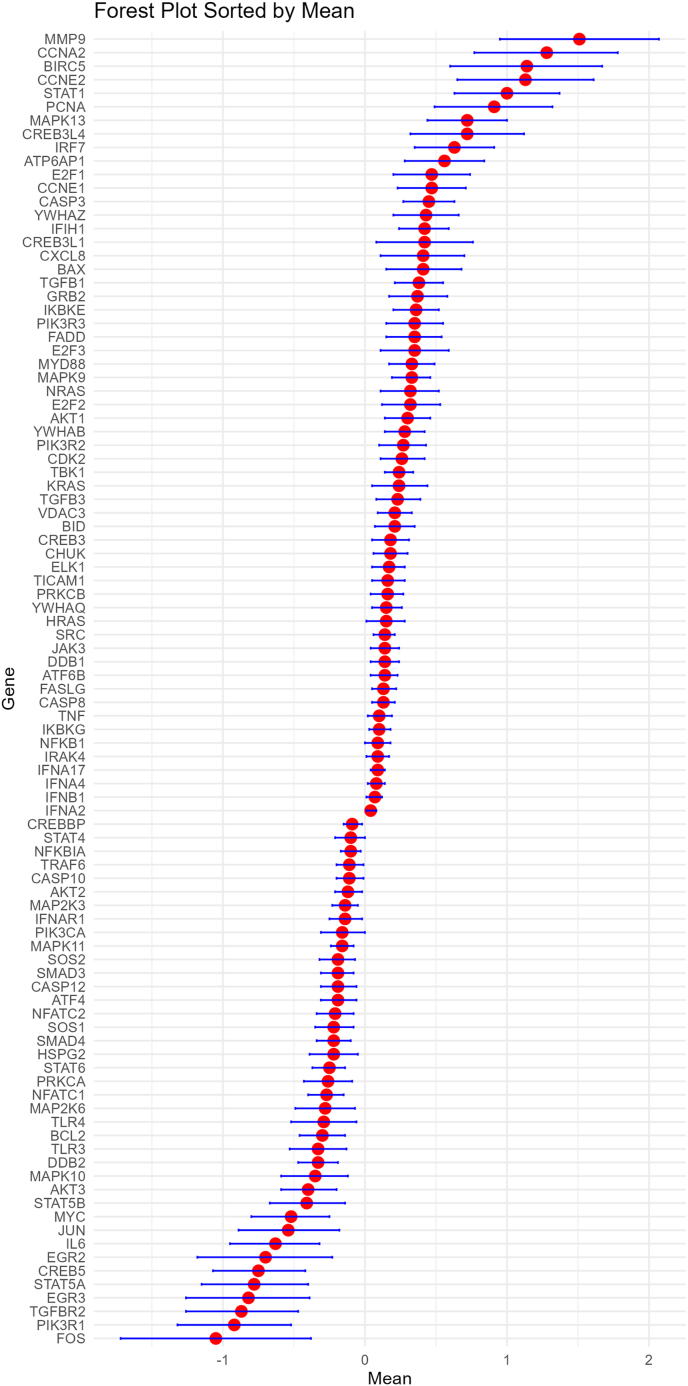
Meta analysis of Hepatitis B virus-associated differentially expressed genes genes between breast cancer tissues and normal tissues.

Meta analysis results showed that the expression levels of 58 genes were higher in breast cancer tissues than those in normal tissues, and the expression levels of 39 genes were lower in breast cancer tissues than those in normal tissues (Fig. 1).

To determine the function of the identified DEGs, functional and pathway enrichment analyses were performed using DAVID. GO enrichment analysis for assessing biological processes revealed that the DEGs were mainly involved in regulation of cell population proliferation, cell surface receptor signaling pathway via JAK-STAT, positive regulation of inflammatory response, cytokine-mediated signaling pathway, positive regulation of apoptotic process. KEGG pathway analysis found that the DEGs were mainly involved in the toll-like receptor signaling pathway, MAPK signaling pathway, TNF signaling pathway, JAK-STAT signaling pathway, PD-L1 expression and PD-1 checkpoint pathway in cancer, T cell receptor signaling pathway, FoxO signaling pathway and apoptosis (Table S3–4). These DEGs might be also involved in ErbB signaling pathway, Estrogen signaling pathway and breast cancer pathways, such as PI3K-Akt signaling pathway (Table S3–4).

### 3.2 Survival analysis for hepatitis B virus-associated genes in breast cancer (supplementary materials)

**Fig. 2:**
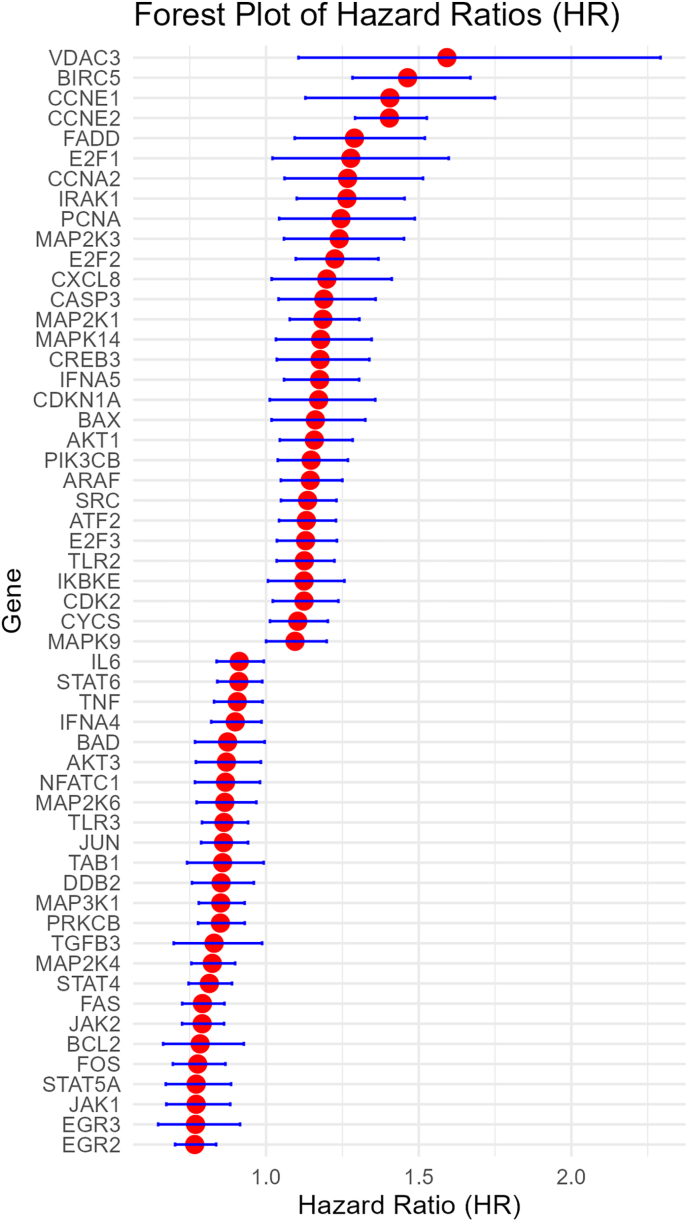
Meta analysis of the relationship of Hepatitis B virus-associated genes with overall survival.

**Fig. 3:**
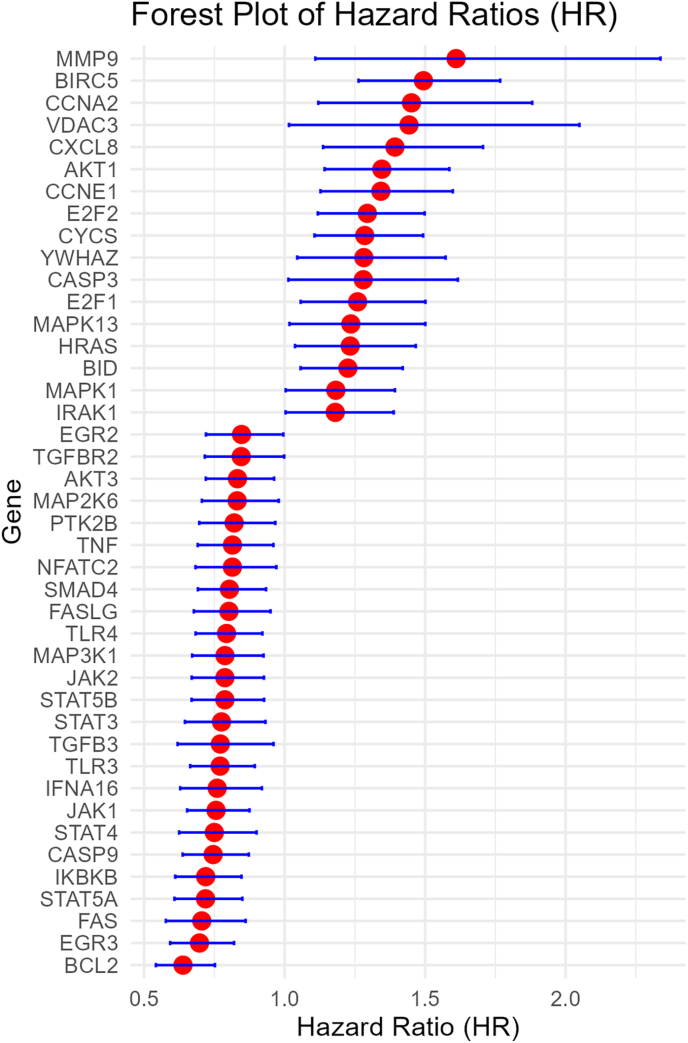
Meta analysis of the relationship of Hepatitis B virus-associated genes with disease free survival.

**Fig. 4:**
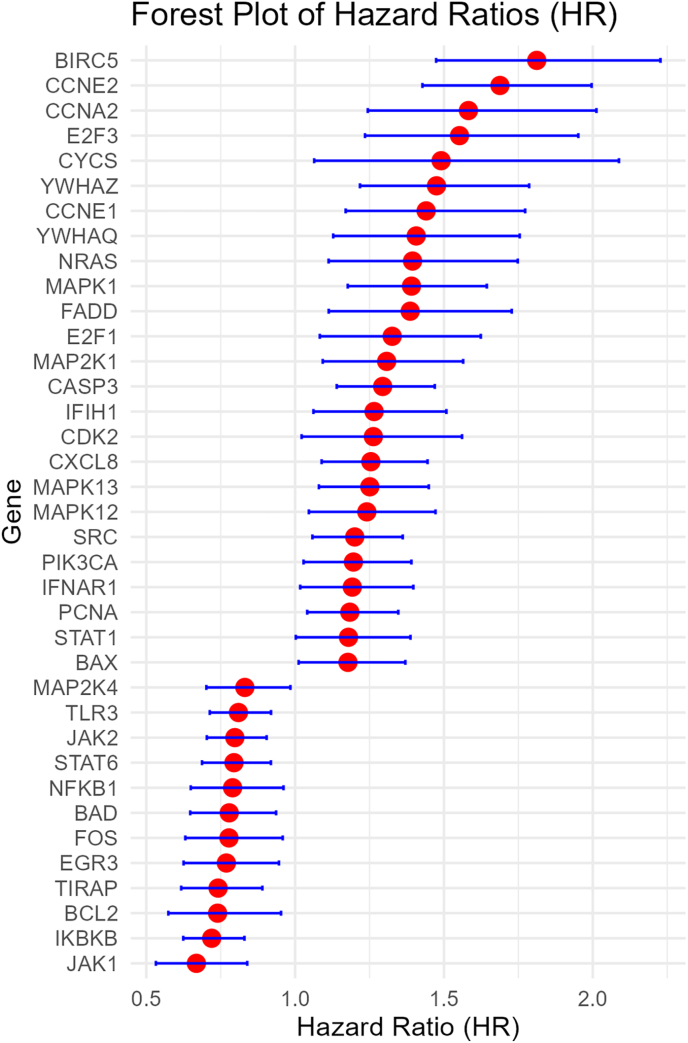
Meta analysis of the relationship of Hepatitis B virus-associated genes with distant metastasis free survival.

**Fig. 5:**
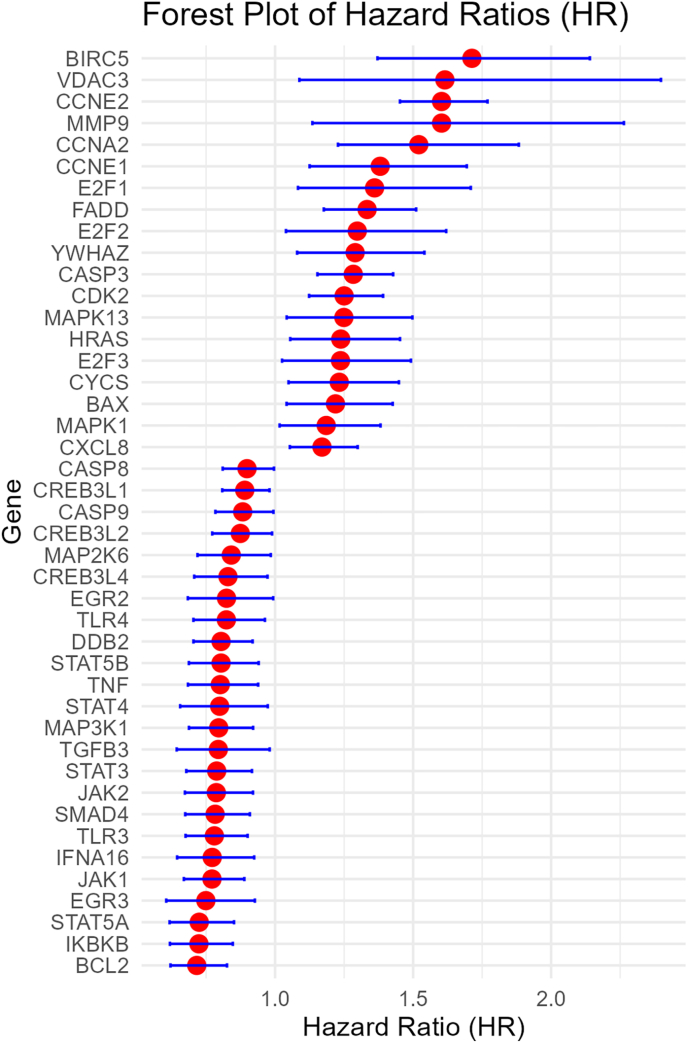
Meta analysis of the relationship of Hepatitis B virus-associated genes with recurrence free survival.

Higher expression of 30 genes were associated with worse OS, while higher expression of 25 genes were associated with better OS (Fig. 2). Higher expression of 17 genes were associated with worse DFS, while higher expression of 24 genes were associated with better DFS (Fig. 3). Higher expression of 25 genes were associated with worse DMFS, while higher expression of 12 genes were associated with better DMFS (Fig. 4). Higher expression of 19 genes were associated with worse RFS, while higher expression of 28 genes were associated with better RFS (Fig. 5).

### 3.3 Comparison between meta analysis methods and databases merging methods

We used KMplot and bc-GenExMiner v5.1 to validate our results for 106 genes, none of which were associated with either improved or reduced overall survival (OS) based on meta analysis results (Table S5). The meta analysis results of 44 genes were similar to those in KMplot and there were 40 genes with similar results to bc-GenExMiner v5.1. Among 31 genes that were associated with worse OS, 26 genes were validated by bc-GenExMiner v5.1, and 22 genes were validated by KM plotter, 19 genes were validated by both bc-GenExMiner v5.1 and KM plotter. Among 26 genes that were associated with better OS based on our meta analysis results, 24 genes were validated by bc-GenExMiner v5.1, 23 genes were validated by KM plotter, 22 genes were validated by both bc-GenExMiner v5.1 and KM plotter.

### 3.4 Genes that were associated with worse survival in breast cancer

Higher expression of seven genes (BIRC5, CASP3, CCNA2, CCNE1, CXCL8, CYCS, E2F1) were associated with worse survival (including OS, DFS, RFS, DMFS) and higher expression of five genes (BCL2, EGR3, JAK1, JAK2, TLR3) were associated with better survival. For further investigating the significant role of these seven genes, 211 candidate drugs targeting these genes were predicted. Among these drugs, we found tamoxifen, 5-fluorouracil, doxorubicin, fulvestrant, capsaicin, gemcitabine, etoposide, vorinostat and paclitaxel might target three or more genes (Fig. S1). We also identified other drugs that might target these seven genes, including resveratrol, troglitazone, alvocidib, curcumin, ly 294002 and bortezomib.

### 3.5 RNAseq analysis

**Fig. 6:**
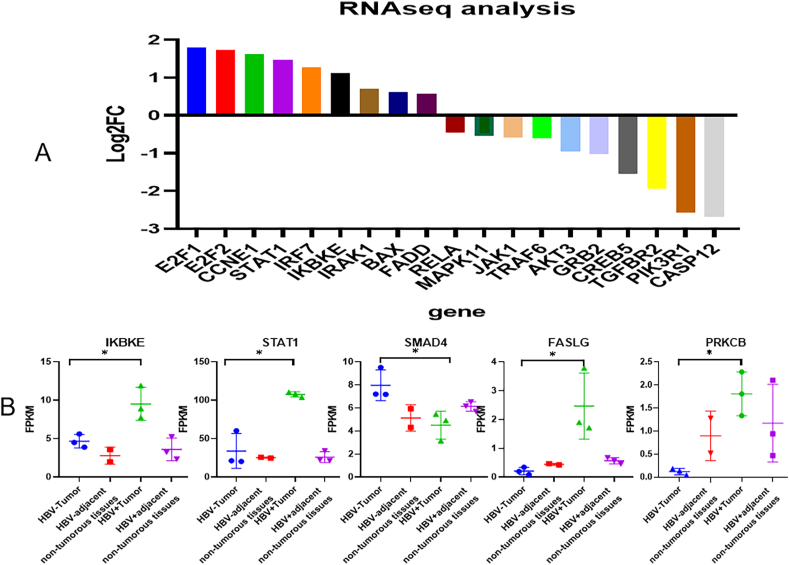
Hepatitis B virus-associated differentially expressed genes genes between breast cancer tissues and normal tissues based on RNAseq analysis.

**Fig. 7:**
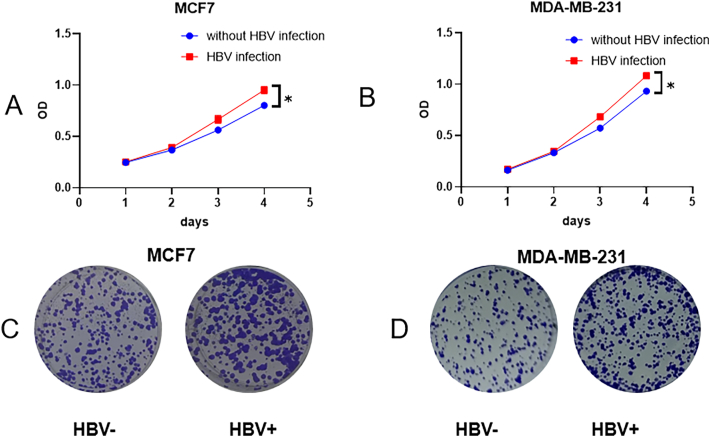
HBV might affect cell proliferation and gene expression in breast cancer patients (A: CCK for MCF7 cells; B:clonal formation assay for MCF7 cells two weeks after seeding; C: CCK for MDA-MB-231 cells; D:clonal formation assay for MDA-MB-231 cells two weeks after seeding).

Our RNA seq analysis also confirmed that the expression levels of eight genes (BAX, CCNE1, E2F1, E2F2, FADD, IKBKE, IRF7, STAT1) were higher in breast cancer tissues than those in normal tissues, and the expression levels of seven genes (AKT3, CASP12, CREB5, MAPK11, PIK3R1, TGFBR2, TRAF6) were lower in breast cancer tissues than those in normal tissues (Fig. 6, Table S6). KEGG pathway analysis revealed that similar enriched pathways were identified across the datasets through meta-analysis (Table S7). Our RNAseq analysis further confirmed that HBV infection might increase the expression of PRKCB, IKBKE, FASLG, STAT1 and decrease the expression of SMAD4 in TNBC tissues (Fig. 7).

### 3.6 In vivo cell experiment

**Fig. 8:**
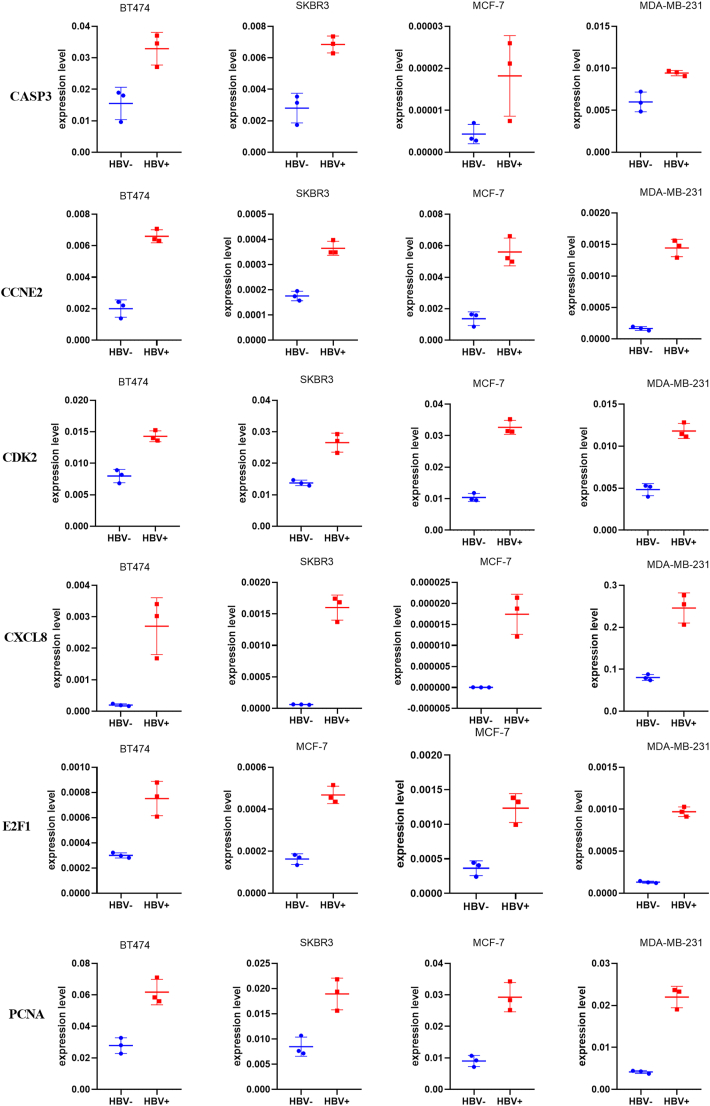
PCR results for Hepatitis B virus related genes.

CCK assays demonstrated that HBV infection enhances cell proliferation. Consistently, colony formation assays confirmed that HBV infection also promotes colony formation. HBV infection lead to increased expression levels of several key genes in breast cancer cell lines, including CDK2, PCNA, CCNE2, CXCL8, E2F1, and CASP3 (Fig. 8). This suggests that HBV could play a role in promoting cell proliferation and survival in these lines. However, the expression levels of other genes following HBV infection were not consistent across different breast cancer cell lines, indicating variability in how different cell types respond to HBV. This variability might reflect differences in cellular context, underlying genetics, or the tumor microenvironment, which could influence the overall disease progression and response to treatment.

## 4 Discussion

Although HBV is primarily associated with liver cancer, increasing evidence suggests potential links to breast cancer and other types of cancer through various mechanisms [[6], [7], [8],^15^]. HBV is known for its potential to cause cancer, primarily because it can integrate its viral DNA into the host's genome. This integration can activate oncogenes and disrupt the function of tumor suppressor genes [[16], [17], [18]]. The virus can trigger cellular stress responses and influence cell cycle regulation, both of which may contribute to tumorigenesis [19]. HBV infection can alter the tumor microenvironment, making it conducive to tumor growth and metastasis [20,21]. Chronic HBV infection leads to persistent inflammation, increasing the risk of cancer. This heightened risk is associated with factors such as oxidative stress and the release of pro-inflammatory cytokines, both of which can promote tumor growth [22]. Given that breast cancer is often hormone-dependent, any changes in estrogen levels or signaling pathways resulting from HBV infection could potentially impact the proliferation of breast tumor cells [23]. The interaction between HBV infection and hormonal factors in breast tissue may represent a pathway through which HBV infection contributes to the biology of breast cancer.

Some studies have reported that women with chronic HBV infection may be at an increased risk of developing breast cancer [7,15], although the results have been mixed and warrant further investigation. Our study identified 97 genes that were DEGs between breast cancer and normal breast tissues. These genes regulate cell growth, signaling pathways (JAK-STAT, MAPK, PI3K-Akt), inflammatory responses, apoptosis, and immune checkpoints in cancer. These DEGs also play important roles in major regulatory pathways associated with cancer. Specifically, they could be involved in the ErbB signaling pathway, which is essential for cell growth and differentiation, the Estrogen signaling pathway, which is crucial for hormonal regulation and breast tissue development, and broader pathways directly linked to breast cancer pathogenesis. Through these pathways, these DEGs may influence processes such as tumor cell proliferation, survival, metastasis, and response to therapy. Our CCK assays showed that HBV infection leads to increased cell proliferation, indicating that infected cells grow and multiply more rapidly. This finding was further supported by colony formation assays, which demonstrated that HBV infection enhances the ability of cells to form colonies, a hallmark of sustained proliferative capacity. HBV infection increase the expression of CDK2, PCNA, CCNE2, and E2F1, further confirmed the roles of HBV in cell growth. We also confirmed that HBV increase the expression of CXCL8, which implied that HBV might affect inflammatory microenvironment. The HBsAg-positive breast cancer patients had significantly inferior DFS and OS as compared with the HBsAg-negative cohort [9,24]. HBsAg has been identified as an independent risk factor for liver metastasis, with patients who are HBsAg-positive experiencing significantly shorter liver metastasis-free survival (LMFS) compared to those who are HBsAg-negative [25]. No studies are currently available to elucidate how HBV influences breast cancer biology and patient survival. In our study, we identified several genes that may impact the prognosis of breast cancer patients. These genes appear to be involved in processes such as viral carcinogenesis, the p53 signaling pathway, cellular senescence, apoptosis, and cell cycle regulation. In vivo cell experiment, we confirmed that HBV infection may lead to increased expression levels of several key genes in breast cancer cell lines, including CDK2, PCNA, CCNE2, CXCL8, E2F1, and CASP3. CDK2 [26], PCNA [27], CXCL8 [28], and E2F1 [29] were involved in the cell cycle, and were associated with worse survival for breast cancer. Activation of CDK2 by cyclins E1/2 and A results in hyperphosphorylation of Rb, which establishes a positive feedback loop to sustain the expression of essential proteins for S phase, thereby irreversibly committing cells to complete the cell cycle [30]. Proliferating cell nuclear antigen (PCNA) is a key nuclear protein that acts as a processivity factor for DNA polymerase δ, playing a crucial role in DNA replication and repair. As an important marker of cell proliferation, PCNA promotes cancer cell division and breast cancer development, while also inhibiting apoptosis, thereby facilitating tumor progression [31]. This suggests that HBV could play a role in promoting cell proliferation and survival in these lines.

Our study aimed to investigate the relationship and potential mechanisms linking HBV-related genes to breast cancer tumorigenesis and progression. We identified several genes that exhibited differential expression between breast cancer and normal breast tissues, which were also associated with poorer survival outcomes in breast cancer patients. To conduct our analysis, we employed two methods. The first method was a meta-analysis, a powerful statistical technique that integrates findings from multiple independent studies to achieve a comprehensive understanding of a specific research question. Meta-analysis provides a systematic and quantitative approach to aggregating data, enhancing statistical power and yielding more precise estimates of effect sizes. This method analyzed data based on aggregated findings from each study while acknowledging their respective strengths and limitations. The second method involved merging data from various studies to create pooled cohorts, which included a batch effect removal step to ensure the data were comparable. We compared results from these two approaches and identified 102 genes that were not associated with overall survival. Conversely, we found that 38 genes were linked to significant overall survival in bc-GenExMiner v5.1, while 44 genes showed similar associations in KMPlot. These discrepancies suggest that data merging methods may produce false positive results. Consequently, we recommend that all studies using online tools such as KMPlot and bc-GenExMiner v5.1 should be validated through RT-qPCR to ensure reliable results.

Several studies have indicated that certain chemotherapeutic agents, including paclitaxel and epirubicin, may facilitate HBV replication [32,33]. This is an important consideration, especially for patients with chronic HBV infection. The mechanism behind this could involve increased immune suppression and alterations in liver function, making the liver more susceptible to viral activation. While the presence of HBV does not typically dictate the choice of chemotherapy agents, it is crucial that clinicians remain vigilant regarding the potential complications of HBV reactivation during treatment. In clinical practice, monitoring HBV status before and during treatment is recommended to mitigate risks. Patients with HBV might experience altered pharmacokinetics due to liver dysfunction caused by viral activity, potentially impacting the efficacy and toxicity of these therapies. Understanding how HBV interacts with hormone receptors, signaling pathways, and treatment responses is critical but still being explored in clinical studies.

Although our study is the first to investigate the relationship and potential mechanisms of HBV-related genes in breast cancer tumorigenesis and progression using meta-analysis methods, it has several limitations. The prognostic analysis conducted in our study was not validated by independent clinical cohorts, which limits the robustness of our conclusions. Our primary objective was to analyze HBV-related genes in the context of breast cancer tumorigenesis and progression, but we cannot provide conclusive genetic mechanistic evidence. Additionally, we were unable to confirm the direct relationship between HBV and breast cancer. Future research should focus on validating these results through appropriate experimental and clinical studies to establish a clearer understanding of the interplay between HBV and breast cancer. The analyzed data are quite heterogeneous because, in some datasets, the tumour tissue samples are not well defined; in others, they are distinguished into luminal A and B, basal and Her-2 enriched. In some datasets, the control tissues are defined adjacent; in other datasets, they are areas with some mammography density; in others, the control tissues are defined as healthy. It also remains unclear whether the tissue samples retrieved from GEO datasets were from patients infected with hepatitis B, whether HBV replication was active, or whether patients had comorbid conditions that could confound the gene expression profiles.

## 5 Conclusion

HBV-related genes play a role in breast cancer tumorigenesis and progression, which may help explain the mechanisms through which HBV influences breast cancer development and advancement. These genes could be involved in various processes such as cellular signaling pathways, immune responses, and the regulation of cell growth and apoptosis, thereby contributing to the complex interplay between HBV infection and breast cancer pathophysiology. Understanding these interactions may provide valuable insights into potential therapeutic targets and pathways for intervention in HBV-associated breast cancer.

## Ethics approval and consent to participate

The tissues used in this study were obtained from the Second Xiangya Hospital of Central South University with authorized permission. The study was approved by the Ethics Committee of the Second Xiangya Hospital of Central South University (Ethics approval number: K005 and LYF2022183).

## Contribution author-disclosure

LLX, MRZ,: Conceptualisation, data curation, formal analysis, methodology, validation, writing original draft.

HNJ, LJX, SPZ, DHZ, YJ: Investigation, resources, writing review and editing.

LL, MD: Conceptualisation, data curation, formal analysis, methodology, validation, funding acquisition, investigation, resources, supervision, writing original draft.

## Funding

This study was funded by the Changsha Natural Science Foundation (kq2208336), the 10.13039/501100004735Hunan Provincial Natural Science Foundation (2023JJ40831; 2023JJ60441; 2022JJ30863), the 10.13039/501100011790Scientific Research Launch Project for new employees of the Second Xiangya Hospital of Central South University (2022–086), the Science and Technology Innovation Program of Hunan Province (2020SK53410), and National Natural Science Foundation of China (82403234).

## Declaration of competing interest

The authors declare that they have no known competing financial interests or personal relationships that could have appeared to influence the work reported in this paper.

## Figures and Tables

**Figure:**
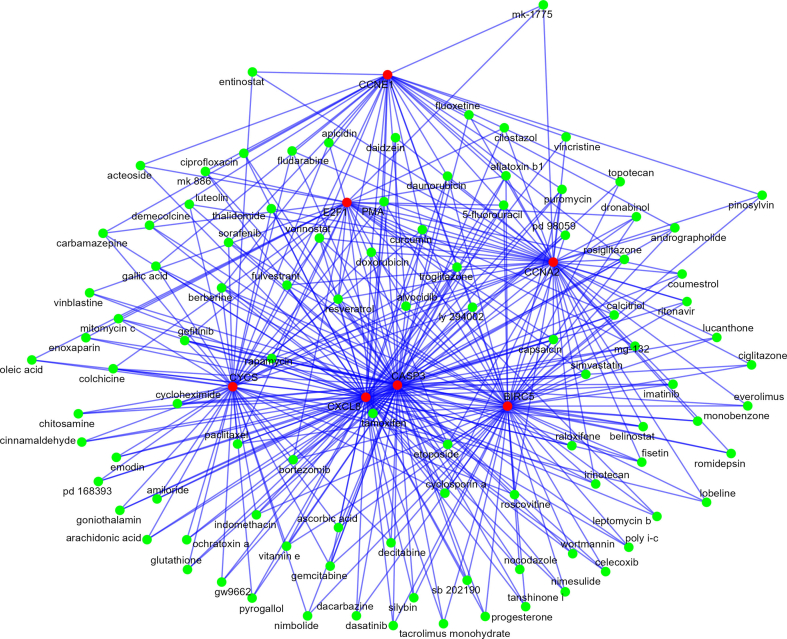


## Data Availability

Data will be made available on request.
