# Supplementary material for: Identification of genes associated with hepatitis B virus infection and breast cancer tumorigenesis and progression
Source: Biochem Biophys Rep. 2025 Jul 14;43:102156. doi: 10.1016/j.bbrep.2025.102156 (PMC12281590; doi:10.1016/j.bbrep.2025.102156)
Supplement: Multimedia component 1 [file mmc1.docx]

**Hepatitis B virus-associated genes between breast cancer and normal tissues**

Meta analysis results showed that the expression levels of 58 genes (AKT1, ATF6B, ATP6AP1, BAX, BID, BIRC5, CASP3, CASP8, CCNA2, CCNE1, CCNE2, CDK2, CHUK, CREB3, CREB3L1, CREB3L4, CXCL8, DDB1, E2F1, E2F2, E2F3, ELK1, FADD, FASLG, GRB2, HRAS, IFIH1, IFNA17, IFNA2, IFNA4, IFNB1, IKBKE, IKBKG, IRAK4, IRF7, JAK3, KRAS, MAPK13, MAPK9, MMP9, MYD88, NFKB1, NRAS, PCNA, PIK3R2, PIK3R3, PRKCB, SRC, STAT1, TBK1, TGFB1, TGFB3, TICAM1, TNF, VDAC3, YWHAB, YWHAQ, YWHAZ) were higher in breast cancer tissues than those in normal tissues, and the expression levels of 39 genes (AKT2, AKT3, ATF4, BCL2, CASP10, CASP12, CREB5, CREBBP, DDB2, EGR2, EGR3, FOS, HSPG2, IFNAR1, IL6, JUN, MAP2K3, MAP2K6, MAPK10, MAPK11, MYC, NFATC1, NFATC2, NFKBIA, PIK3CA, PIK3R1, PRKCA, SMAD3, SMAD4, SOS1, SOS2, STAT4, STAT5A, STAT5B, STAT6, TGFBR2, TLR3, TLR4, TRAF6) were lower in breast cancer tissues than those in normal tissues (Figure 1).

**Overall survival for hepatitis B virus-associated genes in breast cancer**

Higher expression of 30 genes (AKT1, ARAF, ATF2, BAX, BIRC5, CASP3, CCNA2, CCNE1, CCNE2, CDK2, CDKN1A, CREB3, CXCL8, CYCS, E2F1, E2F2, E2F3, FADD, IFNA5, IKBKE, IRAK1, MAP2K1, MAP2K3, MAPK14, MAPK9, PCNA, PIK3CB, SRC, TLR2, VDAC3) were associated with worse OS, while higher expression of 25 genes (AKT3, BAD, BCL2, DDB2, EGR2, EGR3, FAS, FOS, IFNA4, IL6, JAK1, JAK2, JUN, MAP2K4, MAP2K6, MAP3K1, NFATC1, PRKCB, STAT4, STAT5A, STAT6, TAB1, TGFB3, TLR3, TNF) were associated with better OS (Figure 2).

**Disease free survival for hepatitis B virus-associated genes in breast cancer**

Higher expression of 17 genes (AKT1, BID, BIRC5, CASP3, CCNA2, CCNE1, CXCL8, CYCS, E2F1, E2F2, HRAS, IRAK1, MAPK1, MAPK13, MMP9, VDAC3, YWHAZ) were associated with worse DFS, while higher expression of 24 genes (AKT3, BCL2, CASP9, EGR2, EGR3, FAS, FASLG, IFNA16, IKBKB, JAK1, JAK2, MAP2K6, MAP3K1, NFATC2, PTK2B, SMAD4, STAT3, STAT4, STAT5A, STAT5B, TGFB3, TGFBR2, TLR3, TLR4, TNF) were associated with better DFS (Figure 3).

**Distant metastasis free survival for hepatitis B virus-associated genes in breast cancer**

Higher expression of 25 genes (BAX, BIRC5, CASP3, CCNA2, CCNE1, CCNE2, CDK2, CXCL8, CYCS, E2F1, E2F3, FADD, IFIH1, IFNAR1, MAP2K1, MAPK1, MAPK12, MAPK13, NRAS, PCNA, PIK3CA, SRC, STAT1, YWHAQ, YWHAZ) were associated with worse DMFS, while higher expression of 12 genes (BAD, BCL2, EGR3, FOS, IKBKB, JAK1, JAK2, MAP2K4, NFKB1, STAT6, TIRAP, TLR3) were associated with better DMFS (Figure 4).

**Recurrence free survival for hepatitis B virus-associated genes in breast cancer**

Higher expression of 19 genes (BAX, BIRC5, CASP3, CCNA2, CCNE1, CCNE2, CDK2, CXCL8, CYCS, E2F1, E2F2, E2F3, FADD, HRAS, IKBKE, MAPK9, PCNA, SRC, STAT1) were associated with worse RFS, while higher expression of 28 genes (BCL2, CASP8, CASP9, CREB3L1, CREB3L2, CREB3L4, DDB2, EGR2, EGR3, FOS, IKBKB, JAK1, JAK2, JUN, MAP2K4, MAP3K1, MAPK3, NFATC1, PTK2B, SMAD4, STAT3, STAT4, STAT5A, STAT6, TGFB3, TIRAP, TLR3, TRAF6) were associated with better RFS (Figure 5).
