# Supplementary material for: Identification of genes associated with hepatitis B virus infection and breast cancer tumorigenesis and progression
Source: Biochem Biophys Rep. 2025 Jul 14;43:102156. doi: 10.1016/j.bbrep.2025.102156 (PMC12281590; doi:10.1016/j.bbrep.2025.102156)
Supplement: Multimedia component 2 [file mmc2.docx]

Table S1 Characteristics of included datasets

| Databases | Tumor | Control | Tumor | Normal | Country |
| --- | --- | --- | --- | --- | --- |
| GSE10780^[[1](#_ENREF_1)]^ | 42 | 143 | IDC | normal | USA |
| GSE134359^[[2](#_ENREF_2)]^ | 74 | 12 | Tumor | Adjacent normal | Mexico |
| GSE18672^[[3](#_ENREF_3)]^ | 64 | 64 | Tumor | normal | Norway |
| GSE21422^[[4](#_ENREF_4)]^ | 5 | 5 | Tumor | normal | Germany |
| GSE22544^[[4](#_ENREF_4)]^ | 14 | 4 | Tumor | Adjacent normal | USA |
| GSE29044^[[5](#_ENREF_5)]^ | 67 | 36 | Tumor | normal | Saudi Arabia |
| GSE29431 | 54 | 12 | Tumor | normal | Spain |
| GSE33447^[[6](#_ENREF_6)]^ | 8 | 8 | Tumor | normal | China |
| GSE65194^[[7](#_ENREF_7)]^ | 153 | 11 | Tumor | normal | France |
| GSE70905 | 47 | 47 | Tumor | normal | USA |
| GSE70947 | 148 | 148 | Tumor | normal | USA |
| GSE86374 | 124 | 35 | Tumor | Adjacent normal | Mexico |
| TCGA | 1102 | 113 | Tumor | normal | USA |
| GSE36295^[[8](#_ENREF_8)]^ | 45 | 5 | Tumor | normal | Saudi Arabia |
| GSE15852^[[9](#_ENREF_9)]^ | 41 | 43 | Tumor | normal | Malaysia |
| GSE14999^[[10](#_ENREF_10)]^ | 68 | 61 | Tumor | Adjacent normal | Italy |
| GSE31589^[[11](#_ENREF_11)]^ | 12 | 62 | Tumor | Adjacent normal | USA |
| GSE42568^[[12](#_ENREF_12)]^ | 104 | 17 | Tumor | normal | Ireland |
| GSE71053^[[13](#_ENREF_13)]^ | 9 | 9 | Tumor | Adjacent normal | Denmark |
| GSE109169^[[14](#_ENREF_14)]^ | 25 | 25 | Tumor | Adjacent normal | China |

Table S2 Characteristics of included datasets

| Datasets | Country | Treatment | Outcomes | Sample size | Sub-types |
| --- | --- | --- | --- | --- | --- |
| GSE1378^[[15](#_ENREF_15)]^ | USA | tamoxifen | DFS | 60 | ER |
| GSE1456^[[16](#_ENREF_16)]^ | Sweden |  | OS, RFS | 159 | ER,PR,HER2 |
| GSE2034^[[17](#_ENREF_17)]^ | USA |  | DMFS | 286 | ER,PR,HER2 |
| GSE2603^[[18](#_ENREF_18)]^ | USA |  | DMFS | 82 | Her2 |
| GSE3143^[[19](#_ENREF_19)]^ | USA |  | OS | 158 |  |
| GSE3494^[[20](#_ENREF_20)]^ | Singapore |  | DSS | 251 |  |
| GSE4922^[[21](#_ENREF_21)]^ | Singapore | endocrine therapy,systemic therapy | DSS,DFS | 289 | ER |
| GSE5327^[[22](#_ENREF_22)]^ | USA |  | DFS | 58 |  |
| GSE6532^[[23](#_ENREF_23)]^ | Belgium |  | DMFS,RFS | 327 | TN, ER,PR |
| GSE7378^[[24](#_ENREF_24)]^ | USA |  | DFS | 54 | N, ER |
| GSE7390^[[25](#_ENREF_25)]^ | Canada |  | DMFS,RFS,OS | 198 | TN, ER |
| GSE7849^[[26](#_ENREF_26)]^ | USA |  | DFS | 78 | TN, ER,PR |
| GSE9195^[[15](#_ENREF_15)]^ | Belgium | tamoxifen | DMFS,RFS | 77 | T, ER,PR |
| GSE9893^[[27](#_ENREF_27)]^ | France | tamoxifen | DMFS,RFS,OS | 155 | TN, ER,PR |
| GSE11121^[[28](#_ENREF_28)]^ | Germany |  | DMFS | 200 | TN |
| GSE12093^[[29](#_ENREF_29)]^ | USA | tamoxifen | DFS | 136 |  |
| GSE16446^[[30](#_ENREF_30)]^ | Canada | neoadjuvant Anthracyclines | DMFS,OS | 120 | TN,ER,HER2 |
| GSE17705^[[31](#_ENREF_31)]^ | USA | tamoxifen | DRFS | 298 | N, ER |
| GSE17907^[[32](#_ENREF_32)]^ | France |  | MFS | 109 | TN,ER,PR,HER2 |
| GSE19615^[[33](#_ENREF_33)]^ | USA | chemotherapy | DRFS | 115 | N,ER,PR |
| GSE20685^[[34](#_ENREF_34)]^ | China |  | OS | 327 | TN |
| GSE20711^[[35](#_ENREF_35)]^ | Canada |  | OS,RFS | 90 | TN, ER,PR,HER2 |
| GSE21653^[[36](#_ENREF_36)]^ | France |  | DFS | 266 | TN, ER,PR,HER2 |
| GSE22219^[[37](#_ENREF_37)]^ | UK |  | DRFS | 216 | TN,ER |
| GSE25055^[[38](#_ENREF_38)]^ | USA |  | DRFS | 310 | TN,ER,PR,HER2 |
| GSE25065^[[38](#_ENREF_38)]^ | USA |  | DRFS | 198 | TN,ER,PR,HER2 |
| GSE42568^[[12](#_ENREF_12)]^ | Ireland |  | OS, RFS | 121 | T,ER |
| GSE43615 | Italy |  | DFS | 86 | ER |
| GSE45255^[[39](#_ENREF_39)]^ | USA |  | DFS,DMFS | 139 | TN, ER,PR,HER2 |
| GSE48390^[[40](#_ENREF_40)]^ | China |  | DFS | 81 | ER,PR,HER2 |
| GSE58812^[[41](#_ENREF_41)]^ | France |  | OS, MFS | 107 | ER,PR,HER2 |
| GSE58984 | Belgium |  | DMFS | 94 | TN, ER,PR |
| GSE61304^[[42](#_ENREF_42)]^ | Singapore |  | DFS,DMFS | 62 | TN, ER,PR |
| GSE69031^[[43](#_ENREF_43)]^ | USA |  | OS,RFS,DRFS | 130 | TN, ER,PR |
| GSE88770^[[44](#_ENREF_44)]^ | Belgium |  | OS,DRFS | 117 | ER,PR,HER2 |
| GSE158309^[[45](#_ENREF_45)]^ | Germany |  | DMFS | 461 | TN, ER,PR,HER2 |
| GSE199633 | USA |  | OS, RFS | 637 | TN, ER,PR,HER2 |
| METABRIC | Canada,UK |  | OS, RFS | 1979 | TN, ER,PR,HER2 |
| TCGA | USA |  | OS,DFS | 1081 | TN, ER,PR,HER2 |
| GSE25307^[[46](#_ENREF_46)]^ | Sweden |  | OS | 577 | ER,PR |
| GSE26338^[[47](#_ENREF_47)]^ | USA |  | OS, RFS | 100 |  |
| GSE37181^[[48](#_ENREF_48)]^ | Italy |  | DMFS | 123 | T, ER,HER2 |
| GSE45725^[[49](#_ENREF_49)]^ | Canada |  | RFS | 340 | TN,ER,PR,HER2 |
| GSE46563^[[50](#_ENREF_50)]^ | Norway |  | DMFS | 94 | T, ER,HER2 |
| GSE53031^[[51](#_ENREF_51)]^ | Belgium |  | RFS | 167 | T, ER,HER2 |
| GSE86166^[[52](#_ENREF_52)]^ | USA |  | OS, RFS | 366 | ER,PR |
| GSE115577^[[53](#_ENREF_53)]^ | USA |  | RFS,DRFS | 1577 | ER,PR,HER2 |
| GSE135565^[[54](#_ENREF_54)]^ | South Korea |  | OS | 84 | TNBC |
| GSE146558^[[55](#_ENREF_55)]^ | China |  | RFS | 109 | ER,PR |
| GSE162228^[[55](#_ENREF_55)]^ | China |  | OS, RFS | 133 | ER,PR |
| GSE22133^[[56](#_ENREF_56)]^ | Sweden |  | OS | 356 | ER,PR,HER2 |
| GSE22226^[[57](#_ENREF_57)]^ | USA |  | OS,RFS | 150 | ER,PR |
| GSE18229^[[58](#_ENREF_58)]^ | USA |  | OS, RFS | 199 | ER,PR,HER2 |
| GSE19536^[59]^ | Norway |  | DFS | 215 | ER,PR |

Table S3 Biological processes enrichment analysis for differentiated expressed genes between breast cancer and normal breast tissues

| Term | Count | PValue |
| --- | --- | --- |
| GO:0045944~positive regulation of transcription by RNA polymerase II | 21 | 1.15E-14 |
| GO:0006357~regulation of transcription by RNA polymerase II | 16 | 1.09E-07 |
| GO:0045893~positive regulation of DNA-templated transcription | 13 | 5.15E-09 |
| GO:0007165~signal transduction | 12 | 1.43E-05 |
| GO:0016310~phosphorylation | 10 | 2.41E-06 |
| GO:0006355~regulation of DNA-templated transcription | 10 | 2.78E-05 |
| GO:0010628~positive regulation of gene expression | 9 | 4.59E-06 |
| GO:0000122~negative regulation of transcription by RNA polymerase II | 9 | 4.13E-04 |
| GO:0042127~regulation of cell population proliferation | 8 | 1.15E-08 |
| GO:0019221~cytokine-mediated signaling pathway | 7 | 4.78E-07 |
| GO:0006366~transcription by RNA polymerase II | 7 | 5.37E-06 |
| GO:0009410~response to xenobiotic stimulus | 7 | 8.38E-06 |
| GO:0050729~positive regulation of inflammatory response | 6 | 2.13E-06 |
| GO:0043065~positive regulation of apoptotic process | 6 | 3.62E-04 |
| GO:0006954~inflammatory response | 6 | 1.23E-03 |
| GO:0035556~intracellular signal transduction | 6 | 1.36E-03 |
| GO:0043066~negative regulation of apoptotic process | 6 | 2.74E-03 |
| GO:0060395~SMAD protein signal transduction | 5 | 1.69E-07 |
| GO:0007259~cell surface receptor signaling pathway via JAK-STAT | 5 | 1.96E-06 |
| GO:0034644~cellular response to UV | 5 | 4.29E-06 |
| GO:1902895~positive regulation of miRNA transcription | 5 | 5.27E-06 |
| GO:0008286~insulin receptor signaling pathway | 5 | 1.04E-05 |
| GO:0001938~positive regulation of endothelial cell proliferation | 5 | 1.09E-05 |
| GO:0098586~cellular response to virus | 5 | 3.15E-05 |
| GO:0007179~transforming growth factor beta receptor signaling pathway | 5 | 4.01E-05 |
| GO:0006974~DNA damage response | 5 | 2.80E-03 |
| GO:0006915~apoptotic process | 5 | 2.66E-02 |
| GO:0032735~positive regulation of interleukin-12 production | 4 | 8.26E-05 |
| GO:0043434~response to peptide hormone | 4 | 9.44E-05 |
| GO:0043536~positive regulation of blood vessel endothelial cell migration | 4 | 1.21E-04 |
| GO:0048661~positive regulation of smooth muscle cell proliferation | 4 | 1.53E-04 |
| GO:0010718~positive regulation of epithelial to mesenchymal transition | 4 | 1.70E-04 |
| GO:0090398~cellular senescence | 4 | 1.79E-04 |
| GO:0007254~JNK cascade | 4 | 2.19E-04 |
| GO:0050853~B cell receptor signaling pathway | 4 | 2.19E-04 |
| GO:0002931~response to ischemia | 4 | 3.03E-04 |
| GO:0071560~cellular response to transforming growth factor beta stimulus | 4 | 3.16E-04 |
| GO:0007565~female pregnancy | 4 | 4.89E-04 |
| GO:0006952~defense response | 4 | 5.83E-04 |
| GO:0032755~positive regulation of interleukin-6 production | 4 | 1.02E-03 |
| GO:0032760~positive regulation of tumor necrosis factor production | 4 | 1.30E-03 |
| GO:0071456~cellular response to hypoxia | 4 | 2.56E-03 |
| GO:0045766~positive regulation of angiogenesis | 4 | 3.78E-03 |
| GO:0001666~response to hypoxia | 4 | 4.75E-03 |
| GO:0071222~cellular response to lipopolysaccharide | 4 | 5.87E-03 |
| GO:0001701~in utero embryonic development | 4 | 8.76E-03 |
| GO:0043123~positive regulation of canonical NF-kappaB signal transduction | 4 | 9.42E-03 |
| GO:0006468~protein phosphorylation | 4 | 3.54E-02 |
| GO:0031098~stress-activated protein kinase signaling cascade | 3 | 7.54E-05 |
| GO:0033028~myeloid cell apoptotic process | 3 | 1.29E-04 |
| GO:0060397~growth hormone receptor signaling pathway via JAK-STAT | 3 | 1.61E-04 |
| GO:0033033~negative regulation of myeloid cell apoptotic process | 3 | 1.96E-04 |
| GO:0038066~p38MAPK cascade | 3 | 4.26E-04 |
| GO:0051770~positive regulation of nitric-oxide synthase biosynthetic process | 3 | 4.26E-04 |
| GO:0035994~response to muscle stretch | 3 | 6.05E-04 |
| GO:0032924~activin receptor signaling pathway | 3 | 7.41E-04 |
| GO:0038061~non-canonical NF-kappaB signal transduction | 3 | 8.90E-04 |
| GO:0051403~stress-activated MAPK cascade | 3 | 9.70E-04 |
| GO:0038095~Fc-epsilon receptor signaling pathway | 3 | 9.70E-04 |
| GO:0140467~integrated stress response signaling | 3 | 9.70E-04 |
| GO:0040014~regulation of multicellular organism growth | 3 | 9.70E-04 |
| GO:0001782~B cell homeostasis | 3 | 1.14E-03 |
| GO:0034142~toll-like receptor 4 signaling pathway | 3 | 1.52E-03 |
| GO:0048009~insulin-like growth factor receptor signaling pathway | 3 | 1.73E-03 |
| GO:1900017~positive regulation of cytokine production involved in inflammatory response | 3 | 1.73E-03 |
| GO:0045671~negative regulation of osteoclast differentiation | 3 | 1.84E-03 |
| GO:0042267~natural killer cell mediated cytotoxicity | 3 | 1.95E-03 |
| GO:0031663~lipopolysaccharide-mediated signaling pathway | 3 | 1.95E-03 |
| GO:0032743~positive regulation of interleukin-2 production | 3 | 2.06E-03 |
| GO:0045931~positive regulation of mitotic cell cycle | 3 | 2.06E-03 |
| GO:0040018~positive regulation of multicellular organism growth | 3 | 2.18E-03 |
| GO:0043029~T cell homeostasis | 3 | 2.43E-03 |
| GO:0008625~extrinsic apoptotic signaling pathway via death domain receptors | 3 | 2.69E-03 |
| GO:0033077~T cell differentiation in thymus | 3 | 2.69E-03 |
| GO:0060048~cardiac muscle contraction | 3 | 2.96E-03 |
| GO:0032722~positive regulation of chemokine production | 3 | 3.10E-03 |
| GO:0001658~branching involved in ureteric bud morphogenesis | 3 | 3.25E-03 |
| GO:0071364~cellular response to epidermal growth factor stimulus | 3 | 3.39E-03 |
| GO:0030316~osteoclast differentiation | 3 | 3.39E-03 |
| GO:0030890~positive regulation of B cell proliferation | 3 | 3.54E-03 |
| GO:0120163~negative regulation of cold-induced thermogenesis | 3 | 3.85E-03 |
| GO:0014823~response to activity | 3 | 4.17E-03 |
| GO:0070371~ERK1 and ERK2 cascade | 3 | 4.51E-03 |
| GO:0097191~extrinsic apoptotic signaling pathway | 3 | 4.68E-03 |
| GO:0007249~canonical NF-kappaB signal transduction | 3 | 4.68E-03 |
| GO:0071363~cellular response to growth factor stimulus | 3 | 5.58E-03 |
| GO:0032757~positive regulation of interleukin-8 production | 3 | 6.75E-03 |
| GO:0032731~positive regulation of interleukin-1 beta production | 3 | 6.96E-03 |
| GO:0043525~positive regulation of neuron apoptotic process | 3 | 6.96E-03 |
| GO:0009636~response to toxic substance | 3 | 9.89E-03 |
| GO:0006919~activation of cysteine-type endopeptidase activity involved in apoptotic process | 3 | 1.01E-02 |
| GO:0071333~cellular response to glucose stimulus | 3 | 1.01E-02 |
| GO:0030183~B cell differentiation | 3 | 1.30E-02 |
| GO:0032869~cellular response to insulin stimulus | 3 | 1.41E-02 |
| GO:0051091~positive regulation of DNA-binding transcription factor activity | 3 | 1.41E-02 |
| GO:0034976~response to endoplasmic reticulum stress | 3 | 1.47E-02 |
| GO:0030308~negative regulation of cell growth | 3 | 2.24E-02 |
| GO:0000165~MAPK cascade | 3 | 2.45E-02 |
| GO:0001649~osteoblast differentiation | 3 | 2.59E-02 |
| GO:0071356~cellular response to tumor necrosis factor | 3 | 2.82E-02 |
| GO:0032496~response to lipopolysaccharide | 3 | 2.93E-02 |
| GO:0018105~peptidyl-serine phosphorylation | 3 | 3.67E-02 |
| GO:0000209~protein polyubiquitination | 3 | 4.60E-02 |

Table S4 KEGG enrichment analysis for differentiated expressed genes between breast cancer and normal breast tissues

| Term | Count | PValue |
| --- | --- | --- |
| hsa05161:Hepatitis B | 39 | 1.25E-68 |
| hsa05200:Pathways in cancer | 25 | 1.37E-20 |
| hsa05166:Human T-cell leukemia virus 1 infection | 21 | 1.02E-22 |
| hsa05417:Lipid and atherosclerosis | 19 | 1.01E-19 |
| hsa04935:Growth hormone synthesis, secretion and action | 17 | 1.07E-20 |
| hsa05167:Kaposi sarcoma-associated herpesvirus infection | 17 | 2.60E-17 |
| hsa05170:Human immunodeficiency virus 1 infection | 17 | 9.93E-17 |
| hsa04933:AGE-RAGE signaling pathway in diabetic complications | 16 | 3.09E-20 |
| hsa04620:Toll-like receptor signaling pathway | 16 | 1.03E-19 |
| hsa05135:Yersinia infection | 16 | 4.10E-18 |
| hsa05169:Epstein-Barr virus infection | 16 | 1.46E-15 |
| hsa05163:Human cytomegalovirus infection | 16 | 7.49E-15 |
| hsa04010:MAPK signaling pathway | 16 | 4.93E-13 |
| hsa05210:Colorectal cancer | 15 | 2.12E-19 |
| hsa04668:TNF signaling pathway | 15 | 2.12E-17 |
| hsa04926:Relaxin signaling pathway | 15 | 7.61E-17 |
| hsa05162:Measles | 15 | 1.99E-16 |
| hsa04380:Osteoclast differentiation | 15 | 2.70E-16 |
| hsa04630:JAK-STAT signaling pathway | 15 | 3.05E-15 |
| hsa05207:Chemical carcinogenesis - receptor activation | 15 | 9.59E-14 |
| hsa05132:Salmonella infection | 15 | 8.13E-13 |
| hsa05220:Chronic myeloid leukemia | 14 | 2.59E-18 |
| hsa05235:PD-L1 expression and PD-1 checkpoint pathway in cancer | 14 | 2.23E-17 |
| hsa04659:Th17 cell differentiation | 14 | 2.65E-16 |
| hsa04151:PI3K-Akt signaling pathway | 14 | 1.57E-09 |
| hsa05142:Chagas disease | 13 | 6.81E-15 |
| hsa04625:C-type lectin receptor signaling pathway | 13 | 8.72E-15 |
| hsa04722:Neurotrophin signaling pathway | 13 | 4.50E-14 |
| hsa04660:T cell receptor signaling pathway | 13 | 5.52E-14 |
| hsa04068:FoxO signaling pathway | 13 | 1.44E-13 |
| hsa04218:Cellular senescence | 13 | 1.17E-12 |
| hsa05171:Coronavirus disease - COVID-19 | 13 | 1.60E-10 |
| hsa04012:ErbB signaling pathway | 12 | 3.71E-14 |
| hsa04210:Apoptosis | 12 | 6.42E-12 |
| hsa05226:Gastric cancer | 12 | 1.89E-11 |
| hsa05225:Hepatocellular carcinoma | 12 | 7.39E-11 |
| hsa04664:Fc epsilon RI signaling pathway | 11 | 1.70E-13 |
| hsa04917:Prolactin signaling pathway | 11 | 2.29E-13 |
| hsa04662:B cell receptor signaling pathway | 11 | 3.01E-12 |
| hsa04658:Th1 and Th2 cell differentiation | 11 | 3.36E-12 |
| hsa05215:Prostate cancer | 11 | 6.42E-12 |
| hsa01522:Endocrine resistance | 11 | 7.12E-12 |
| hsa04915:Estrogen signaling pathway | 11 | 2.17E-10 |
| hsa05160:Hepatitis C | 11 | 8.18E-10 |
| hsa05164:Influenza A | 11 | 1.77E-09 |
| hsa05205:Proteoglycans in cancer | 11 | 9.25E-09 |
| hsa05203:Viral carcinogenesis | 11 | 9.70E-09 |
| hsa04024:cAMP signaling pathway | 11 | 2.47E-08 |
| hsa05208:Chemical carcinogenesis - reactive oxygen species | 11 | 2.47E-08 |
| hsa05131:Shigellosis | 11 | 6.20E-08 |
| hsa05223:Non-small cell lung cancer | 10 | 1.45E-11 |
| hsa05231:Choline metabolism in cancer | 10 | 2.39E-10 |
| hsa05145:Toxoplasmosis | 10 | 6.72E-10 |
| hsa05418:Fluid shear stress and atherosclerosis | 10 | 5.69E-09 |
| hsa04936:Alcoholic liver disease | 10 | 6.86E-09 |
| hsa05224:Breast cancer | 10 | 8.73E-09 |
| hsa04932:Non-alcoholic fatty liver disease | 10 | 1.47E-08 |
| hsa05152:Tuberculosis | 10 | 4.83E-08 |
| hsa04621:NOD-like receptor signaling pathway | 10 | 7.37E-08 |
| hsa04510:Focal adhesion | 10 | 1.36E-07 |
| hsa05165:Human papillomavirus infection | 10 | 8.50E-06 |
| hsa05168:Herpes simplex virus 1 infection | 10 | 2.84E-04 |
| hsa05221:Acute myeloid leukemia | 9 | 3.27E-10 |
| hsa05212:Pancreatic cancer | 9 | 9.06E-10 |
| hsa01521:EGFR tyrosine kinase inhibitor resistance | 9 | 1.24E-09 |
| hsa05222:Small cell lung cancer | 9 | 4.17E-09 |
| hsa04912:GnRH signaling pathway | 9 | 4.17E-09 |
| hsa04066:HIF-1 signaling pathway | 9 | 1.48E-08 |
| hsa04725:Cholinergic synapse | 9 | 2.27E-08 |
| hsa04217:Necroptosis | 9 | 2.84E-07 |
| hsa04310:Wnt signaling pathway | 9 | 5.67E-07 |
| hsa05415:Diabetic cardiomyopathy | 9 | 1.96E-06 |
| hsa05022:Pathways of neurodegeneration - multiple diseases | 9 | 8.56E-04 |
| hsa05213:Endometrial cancer | 8 | 4.39E-09 |
| hsa05211:Renal cell carcinoma | 8 | 1.49E-08 |
| hsa05214:Glioma | 8 | 2.67E-08 |
| hsa04931:Insulin resistance | 8 | 3.29E-07 |
| hsa04071:Sphingolipid signaling pathway | 8 | 7.10E-07 |
| hsa04650:Natural killer cell mediated cytotoxicity | 8 | 1.09E-06 |
| hsa04728:Dopaminergic synapse | 8 | 1.21E-06 |
| hsa04550:Signaling pathways regulating pluripotency of stem cells | 8 | 2.18E-06 |
| hsa04062:Chemokine signaling pathway | 8 | 1.51E-05 |
| hsa05130:Pathogenic Escherichia coli infection | 8 | 2.09E-05 |
| hsa04015:Rap1 signaling pathway | 8 | 2.77E-05 |
| hsa04014:Ras signaling pathway | 8 | 5.81E-05 |
| hsa05020:Prion disease | 8 | 1.54E-04 |
| hsa05206:MicroRNAs in cancer | 8 | 3.13E-04 |
| hsa05010:Alzheimer disease | 8 | 1.21E-03 |

Table S5 Comparison between meta analysis methods and databases merging methods

|  | overall survival (bc-GenExMiner v5.1) | | | overall survival (meta analysis) | | | overall survival (KM) | | |
| --- | --- | --- | --- | --- | --- | --- | --- | --- | --- |
|  | HR | Lower | Upper | HR | Lower | Upper | HR | Lower | Upper |
| AKT1 | 1.19 | 1.1 | 1.29 | 1.16 | 1.05 | 1.28 | 1.24 | 1.01 | 1.53 |
| AKT2 | 1.15 | 1.05 | 1.27 | 0.97 | 0.85 | 1.12 | 0.72 | 0.52 | 0.98 |
| AKT3 | 0.91 | 0.84 | 0.99 | 0.87 | 0.77 | 0.98 | 0.69 | 0.52 | 0.92 |
| APAF1 | 1.1 | 1.02 | 1.2 | 1.08 | 0.99 | 1.17 | 1.16 | 0.94 | 1.45 |
| ARAF | 1.15 | 1.05 | 1.26 | 1.14 | 1.05 | 1.25 | 0.69 | 0.52 | 0.9 |
| ATF2 | 1.15 | 1.04 | 1.26 | 1.13 | 1.04 | 1.23 | 0.57 | 0.43 | 0.76 |
| ATF4 | 1.1 | 1.01 | 1.2 | 1.09 | 0.95 | 1.25 | 1.51 | 1.23 | 1.86 |
| ATF6B | 1.63 | 1.33 | 1.99 | 0.97 | 0.83 | 1.14 | 1.15 | 0.88 | 1.51 |
| ATP6AP1 | 1.26 | 1.04 | 1.52 | 1.05 | 0.84 | 1.32 | 1.24 | 1.02 | 1.5 |
| BAD | 0.7 | 0.56 | 0.87 | 0.87 | 0.77 | 1.00 | 0.77 | 0.64 | 0.93 |
| BAX | 1.17 | 1.07 | 1.29 | 1.16 | 1.02 | 1.33 | 1.26 | 1.04 | 1.52 |
| BCL2 | 0.47 | 0.39 | 0.57 | 0.78 | 0.66 | 0.93 | 0.53 | 0.44 | 0.64 |
| BID | 1.03 | 0.94 | 1.12 | 1.02 | 0.90 | 1.16 | 1.31 | 0.97 | 1.77 |
| BIRC5 | 1.37 | 1.26 | 1.49 | 1.46 | 1.28 | 1.67 | 2.1 | 1.63 | 2.7 |
| BRAF | 0.64 | 0.52 | 0.79 | 1.11 | 0.99 | 1.25 | 0.7 | 0.53 | 0.91 |
| CASP10 | 1.07 | 0.97 | 1.18 | 1.01 | 0.88 | 1.16 | 0.87 | 0.72 | 1.05 |
| CASP12 | 0.86 | 0.77 | 0.97 | 1.01 | 0.84 | 1.21 | 0.77 | 0.56 | 1.06 |
| CASP3 | 1.24 | 1.02 | 1.5 | 1.19 | 1.04 | 1.36 | 1.15 | 0.94 | 1.42 |
| CASP8 | 0.64 | 0.52 | 0.79 | 0.90 | 0.79 | 1.03 | 0.54 | 0.41 | 0.72 |
| CASP9 | 0.56 | 0.46 | 0.68 | 0.92 | 0.80 | 1.07 | 0.64 | 0.49 | 0.84 |
| CCNA2 | 1.62 | 1.34 | 1.95 | 1.27 | 1.06 | 1.51 | 1.83 | 1.49 | 2.26 |
| CCNE1 | 1.83 | 1.52 | 2.21 | 1.41 | 1.13 | 1.75 | 1.84 | 1.52 | 2.22 |
| CCNE2 | 2.3 | 1.69 | 3.13 | 1.40 | 1.29 | 1.53 | 1.64 | 1.35 | 1.99 |
| CDK2 | 1.4 | 1.13 | 1.74 | 1.12 | 1.02 | 1.24 | 1.22 | 1.01 | 1.48 |
| CDKN1A | 1.11 | 1.02 | 1.21 | 1.17 | 1.01 | 1.36 | 1.27 | 1.04 | 1.53 |
| CHUK | 1.13 | 1 | 1.27 | 1.09 | 0.99 | 1.19 | 1.17 | 0.96 | 1.41 |
| CREB1 | 1.14 | 1.03 | 1.25 | 0.93 | 0.79 | 1.10 | 0.57 | 0.43 | 0.76 |
| CREB3 | 1.25 | 1.13 | 1.38 | 1.18 | 1.04 | 1.34 | 1.17 | 0.94 | 1.46 |
| CREB3L1 | 0.94 | 0.87 | 1.02 | 1.04 | 0.95 | 1.13 | 1.16 | 0.96 | 1.41 |
| CREB3L2 | 1.1 | 1.01 | 1.2 | 0.99 | 0.91 | 1.08 | 1.41 | 1.05 | 1.9 |
| CREB3L3 | 1.05 | 0.95 | 1.15 | 0.96 | 0.79 | 1.16 | 1.43 | 1.1 | 1.88 |
| CREB3L4 | 1.07 | 0.97 | 1.16 | 0.90 | 0.77 | 1.04 | 0.7 | 0.52 | 0.93 |
| CREB5 | 0.88 | 0.71 | 1.09 | 0.97 | 0.87 | 1.09 | 1.37 | 1.03 | 1.82 |
| CREBBP | 0.94 | 0.87 | 1.02 | 0.96 | 0.88 | 1.04 | 0.74 | 0.54 | 1.03 |
| CXCL8 | 1.32 | 1.2 | 1.46 | 1.20 | 1.02 | 1.41 | 1.61 | 1.33 | 1.94 |
| CYCS | 0.9 | 0.82 | 0.99 | 1.10 | 1.01 | 1.20 | 1.82 | 1.39 | 2.39 |
| DDB1 | 1.15 | 1.04 | 1.27 | 0.96 | 0.86 | 1.07 | 0.8 | 0.65 | 0.99 |
| DDB2 | 0.76 | 0.62 | 0.94 | 0.85 | 0.76 | 0.96 | 0.79 | 0.65 | 0.95 |
| DDX3X | 0.92 | 0.84 | 1.01 | 1.00 | 0.89 | 1.14 | 0.83 | 0.68 | 1.02 |
| E2F1 | 1.24 | 1.14 | 1.36 | 1.28 | 1.02 | 1.60 | 1.66 | 1.37 | 2.02 |
| E2F2 | 1.39 | 1.27 | 1.53 | 1.23 | 1.10 | 1.37 | 1.47 | 1.05 | 2.07 |
| E2F3 | 1.25 | 1.14 | 1.37 | 1.13 | 1.04 | 1.23 | 1.39 | 1.15 | 1.68 |
| EGR2 | 0.62 | 0.5 | 0.76 | 0.77 | 0.70 | 0.84 | 0.78 | 0.64 | 0.94 |
| EGR3 | 0.57 | 0.46 | 0.69 | 0.77 | 0.65 | 0.91 | 0.67 | 0.55 | 0.83 |
| ELK1 |  |  |  | 1.03 | 0.92 | 1.16 | 0.83 | 0.69 | 1.01 |
| EP300 | 1.09 | 0.99 | 1.19 | 1.00 | 0.89 | 1.13 | 1.18 | 0.98 | 1.43 |
| FADD | 1.56 | 1.37 | 1.78 | 1.29 | 1.09 | 1.52 | 1.65 | 1.35 | 2.01 |
| FAS | 0.7 | 0.58 | 0.85 | 0.79 | 0.73 | 0.86 | 0.65 | 0.54 | 0.79 |
| FASLG | 0.72 | 0.6 | 0.87 | 0.91 | 0.78 | 1.06 | 0.71 | 0.59 | 0.86 |
| FOS | 0.61 | 0.5 | 0.75 | 0.78 | 0.69 | 0.87 | 0.66 | 0.54 | 0.8 |
| GRB2 | 1.18 | 1.08 | 1.29 | 1.00 | 0.89 | 1.13 | 0.79 | 0.61 | 1.04 |
| HRAS | 1.22 | 1.12 | 1.33 | 1.12 | 0.93 | 1.34 | 1.27 | 1.01 | 1.59 |
| HSPG2 | 1.05 | 0.96 | 1.13 | 1.03 | 0.91 | 1.16 | 0.83 | 0.67 | 1.02 |
| IFIH1 | 1.11 | 1.02 | 1.22 | 1.13 | 0.99 | 1.28 | 1.31 | 1.06 | 1.63 |
| IFNA1 | 1.04 | 0.94 | 1.16 | 0.97 | 0.88 | 1.08 | 0.8 | 0.66 | 0.96 |
| IFNA10 | 1.05 | 0.95 | 1.16 | 1.00 | 0.91 | 1.09 | 1.13 | 0.94 | 1.37 |
| IFNA13 |  |  |  | 1.02 | 0.91 | 1.15 | 0.8 | 0.66 | 0.96 |
| IFNA14 | 0.93 | 0.84 | 1.02 | 1.06 | 0.96 | 1.16 | 0.88 | 0.72 | 1.08 |
| IFNA16 | 0.88 | 0.76 | 1.02 | 1.08 | 0.99 | 1.19 | 0.8 | 0.66 | 0.97 |
| IFNA17 | 1.19 | 0.99 | 1.43 | 1.04 | 0.85 | 1.26 | 0.84 | 0.7 | 1.02 |
| IFNA2 | 0.91 | 0.82 | 1.02 | 1.04 | 0.95 | 1.14 | 0.8 | 0.64 | 1 |
| IFNA21 | 1.08 | 0.99 | 1.18 | 0.99 | 0.91 | 1.09 | 0.89 | 0.74 | 1.08 |
| IFNA4 | 0.87 | 0.79 | 0.97 | 0.90 | 0.82 | 0.99 | 0.85 | 0.7 | 1.03 |
| IFNA5 | 1.15 | 1.06 | 1.26 | 1.18 | 1.06 | 1.30 | 0.85 | 0.71 | 1.03 |
| IFNA6 | 0.9 | 0.81 | 1.01 | 1.01 | 0.91 | 1.12 | 0.86 | 0.71 | 1.04 |
| IFNA7 | 0.61 | 0.2 | 1.92 | 1.02 | 0.93 | 1.12 | 0.87 | 0.71 | 1.06 |
| IFNA8 | 0.95 | 0.86 | 1.04 | 0.97 | 0.84 | 1.12 | 0.87 | 0.7 | 1.07 |
| IFNAR1 | 1.13 | 1.02 | 1.24 | 1.07 | 0.92 | 1.25 | 0.68 | 0.52 | 0.89 |
| IFNB1 | 1.09 | 0.99 | 1.2 | 1.02 | 0.94 | 1.12 | 0.89 | 0.74 | 1.08 |
| IKBKB | 0.82 | 0.74 | 0.91 | 0.94 | 0.83 | 1.07 | 0.65 | 0.52 | 0.8 |
| IKBKE | 0.91 | 0.82 | 1.01 | 1.12 | 1.01 | 1.26 | 1.06 | 0.87 | 1.28 |
| IKBKG | 1.09 | 0.93 | 1.27 | 1.06 | 0.96 | 1.18 | 0.86 | 0.7 | 1.06 |
| IL6 | 0.86 | 0.69 | 1.09 | 0.91 | 0.84 | 0.99 | 0.88 | 0.72 | 1.08 |
| IRAK1 | 1.19 | 1.09 | 1.3 | 1.26 | 1.10 | 1.45 | 1.66 | 1.25 | 2.19 |
| IRAK4 | 0.9 | 0.78 | 1.02 | 0.99 | 0.91 | 1.09 | 0.82 | 0.67 | 1 |
| IRF3 | 1.12 | 1.03 | 1.21 | 1.05 | 0.91 | 1.20 | 0.82 | 0.68 | 0.98 |
| IRF7 | 1.21 | 1.11 | 1.31 | 1.11 | 0.94 | 1.30 | 0.87 | 0.7 | 1.07 |
| JAK1 | 0.51 | 0.42 | 0.62 | 0.77 | 0.67 | 0.88 | 0.51 | 0.39 | 0.67 |
| JAK2 | 0.52 | 0.43 | 0.64 | 0.79 | 0.73 | 0.86 | 0.58 | 0.47 | 0.72 |
| JAK3 | 1.07 | 0.98 | 1.16 | 0.99 | 0.86 | 1.13 | 0.74 | 0.56 | 0.99 |
| JUN | 0.75 | 0.62 | 0.91 | 0.86 | 0.79 | 0.94 | 1.11 | 0.9 | 1.35 |
| KRAS | 1.12 | 0.93 | 1.36 | 0.97 | 0.83 | 1.13 | 0.85 | 0.7 | 1.02 |
| MAP2K1 | 1.49 | 1.22 | 1.81 | 1.19 | 1.08 | 1.31 | 1.36 | 1.12 | 1.65 |
| MAP2K2 | 1.09 | 1.01 | 1.18 | 1.10 | 0.97 | 1.23 | 0.89 | 0.73 | 1.07 |
| MAP2K3 | 1.35 | 1.09 | 1.68 | 1.24 | 1.06 | 1.45 | 1.24 | 1.03 | 1.5 |
| MAP2K4 | 0.68 | 0.56 | 0.82 | 0.82 | 0.76 | 0.90 | 0.59 | 0.48 | 0.72 |
| MAP2K6 | 0.72 | 0.58 | 0.9 | 0.87 | 0.77 | 0.97 | 0.78 | 0.63 | 0.96 |
| MAP2K7 | 0.76 | 0.61 | 0.95 | 0.93 | 0.83 | 1.04 | 0.73 | 0.55 | 0.98 |
| MAP3K1 | 0.59 | 0.49 | 0.72 | 0.85 | 0.78 | 0.93 | 0.45 | 0.34 | 0.59 |
| MAP3K7 | 0.88 | 0.71 | 1.09 | 1.08 | 0.95 | 1.22 | 0.78 | 0.64 | 0.96 |
| MAPK1 | 1.21 | 0.98 | 1.49 | 1.13 | 0.98 | 1.31 | 0.82 | 0.61 | 1.09 |
| MAPK10 | 0.72 | 0.59 | 0.88 | 1.05 | 0.96 | 1.15 | 1.16 | 0.88 | 1.52 |
| MAPK11 | 0.74 | 0.59 | 0.92 | 1.01 | 0.91 | 1.13 | 1.13 | 0.93 | 1.37 |
| MAPK12 | 1.47 | 1.19 | 1.82 | 1.08 | 0.99 | 1.17 | 1.28 | 0.97 | 1.68 |
| MAPK13 | 1.12 | 0.9 | 1.38 | 1.08 | 1.00 | 1.17 | 1.26 | 1.04 | 1.52 |
| MAPK14 | 1.31 | 1.09 | 1.58 | 1.18 | 1.03 | 1.35 | 0.82 | 0.67 | 1 |
| MAPK3 | 0.67 | 0.55 | 0.83 | 1.00 | 0.92 | 1.09 | 0.63 | 0.51 | 0.77 |
| MAPK8 | 0.76 | 0.63 | 0.92 | 0.90 | 0.80 | 1.01 | 0.61 | 0.46 | 0.81 |
| MAPK9 | 1.18 | 0.97 | 1.43 | 1.09 | 1.00 | 1.20 | 0.74 | 0.56 | 0.99 |
| MAVS | 0.8 | 0.65 | 1 | 1.05 | 0.96 | 1.14 | 0.8 | 0.61 | 1.04 |
| MMP9 | 1.37 | 1.13 | 1.65 | 0.95 | 0.83 | 1.09 | 1.21 | 0.98 | 1.49 |
| MYC | 0.77 | 0.62 | 0.94 | 1.08 | 0.90 | 1.29 | 1.12 | 0.91 | 1.39 |
| MYD88 | 0.8 | 0.64 | 1.01 | 1.03 | 0.90 | 1.18 | 1.24 | 1.03 | 1.51 |
| NFATC1 | 0.61 | 0.5 | 0.76 | 0.87 | 0.77 | 0.98 | 0.64 | 0.52 | 0.8 |
| NFATC2 | 0.68 | 0.55 | 0.85 | 1.03 | 0.91 | 1.17 | 0.57 | 0.43 | 0.76 |
| NFATC3 | 0.93 | 0.76 | 1.13 | 1.01 | 0.86 | 1.19 | 0.65 | 0.46 | 0.9 |
| NFATC4 | 0.68 | 0.56 | 0.83 | 0.98 | 0.87 | 1.11 | 0.85 | 0.62 | 1.16 |
| NFKB1 | 0.68 | 0.56 | 0.82 | 0.90 | 0.66 | 1.22 | 0.64 | 0.53 | 0.78 |
| NFKBIA | 0.62 | 0.51 | 0.75 | 0.97 | 0.85 | 1.10 | 0.77 | 0.64 | 0.94 |
| NRAS | 0.82 | 0.65 | 1.02 | 0.98 | 0.90 | 1.07 | 0.81 | 0.62 | 1.06 |
| PCNA | 1.51 | 1.25 | 1.84 | 1.25 | 1.04 | 1.49 | 1.48 | 1.19 | 1.83 |
| PIK3CA | 0.76 | 0.61 | 0.95 | 1.05 | 0.95 | 1.17 | 0.73 | 0.59 | 0.9 |
| PIK3CB | 1.4 | 1.16 | 1.7 | 1.15 | 1.04 | 1.27 | 1.27 | 1.05 | 1.54 |
| PIK3CD | 0.75 | 0.6 | 0.95 | 0.92 | 0.84 | 1.00 | 0.59 | 0.46 | 0.74 |
| PIK3R1 | 0.66 | 0.54 | 0.8 | 0.92 | 0.77 | 1.10 | 0.79 | 0.65 | 0.95 |
| PIK3R2 | 0.82 | 0.68 | 0.99 | 0.94 | 0.86 | 1.04 | 0.62 | 0.47 | 0.81 |
| PIK3R3 | 0.7 | 0.6 | 0.87 | 0.96 | 0.85 | 1.10 | 0.87 | 0.72 | 1.06 |
| PRKCA | 0.78 | 0.63 | 0.97 | 1.01 | 0.85 | 1.19 | 1.46 | 1.11 | 1.94 |
| PRKCB | 0.67 | 0.52 | 0.88 | 0.85 | 0.78 | 0.93 | 0.55 | 0.42 | 0.72 |
| PRKCG | 1.18 | 0.98 | 1.43 | 1.07 | 0.99 | 1.17 | 0.87 | 0.65 | 1.16 |
| PTK2B | 0.77 | 0.62 | 0.96 | 0.95 | 0.88 | 1.04 | 0.82 | 0.67 | 0.99 |
| RAF1 | 0.72 | 0.58 | 0.9 | 0.96 | 0.84 | 1.10 | 1.15 | 0.95 | 1.39 |
| RB1 | 0.85 | 0.68 | 1.07 | 1.05 | 0.91 | 1.20 | 1.15 | 0.95 | 1.39 |
| RELA | 0.87 | 0.71 | 1.05 | 1.04 | 0.94 | 1.16 | 0.81 | 0.67 | 0.98 |
| SLC10A1 | 0.91 | 0.73 | 1.14 | 1.05 | 0.96 | 1.14 | 0.86 | 0.7 | 1.07 |
| SMAD3 | 0.63 | 0.52 | 0.77 | 0.97 | 0.84 | 1.13 | 0.77 | 0.64 | 0.93 |
| SMAD4 | 0.66 | 0.53 | 0.82 | 0.89 | 0.79 | 1.01 | 0.68 | 0.52 | 0.9 |
| SOS1 | 0.83 | 0.6 | 1.02 | 1.06 | 0.96 | 1.17 | 0.79 | 0.58 | 1.08 |
| SOS2 | 0.67 | 0.54 | 0.82 | 1.04 | 0.94 | 1.14 | 0.9 | 0.74 | 1.09 |
| SRC | 1.18 | 0.98 | 1.43 | 1.14 | 1.05 | 1.23 | 1.33 | 1.02 | 1.74 |
| STAT1 | 1.3 | 1.05 | 1.62 | 1.16 | 0.89 | 1.49 | 0.77 | 0.61 | 0.95 |
| STAT2 | 0.81 | 0.66 | 1 | 1.04 | 0.87 | 1.25 | 0.7 | 0.53 | 0.92 |
| STAT3 | 0.81 | 0.67 | 0.98 | 0.90 | 0.80 | 1.01 | 0.56 | 0.43 | 0.73 |
| STAT4 | 0.59 | 0.49 | 0.71 | 0.81 | 0.75 | 0.89 | 0.72 | 0.59 | 0.87 |
| STAT5A | 0.6 | 0.5 | 0.73 | 0.77 | 0.67 | 0.89 | 0.63 | 0.51 | 0.78 |
| STAT5B | 0.64 | 0.51 | 0.79 | 0.89 | 0.76 | 1.05 | 0.66 | 0.5 | 0.86 |
| STAT6 | 0.56 | 0.45 | 0.69 | 0.91 | 0.84 | 0.99 | 0.65 | 0.53 | 0.8 |
| TAB1 | 0.66 | 0.53 | 0.81 | 0.86 | 0.74 | 0.99 | 0.82 | 0.68 | 1 |
| TAB2 | 1.3 | 1.04 | 1.63 | 0.93 | 0.84 | 1.02 | 0.83 | 0.68 | 1.01 |
| TBK1 | 0.64 | 0.52 | 0.79 | 1.10 | 0.96 | 1.27 | 0.75 | 0.61 | 0.92 |
| TGFB1 | 0.81 | 0.66 | 1 | 0.98 | 0.89 | 1.07 | 0.68 | 0.56 | 0.83 |
| TGFB2 | 0.81 | 0.65 | 0.99 | 1.03 | 0.91 | 1.15 | 1.21 | 0.98 | 1.48 |
| TGFB3 | 0.59 | 0.48 | 0.72 | 0.83 | 0.70 | 0.99 | 0.57 | 0.45 | 0.72 |
| TGFBR1 | 1.22 | 1 | 1.47 | 1.11 | 0.98 | 1.25 | 1.53 | 1.16 | 2.03 |
| TGFBR2 | 0.72 | 0.59 | 0.87 | 0.93 | 0.80 | 1.07 | 0.65 | 0.54 | 0.79 |
| TICAM1 | 1.35 | 1.09 | 1.68 | 1.08 | 0.93 | 1.25 | 1.17 | 0.94 | 1.47 |
| TIRAP | 0.64 | 0.53 | 0.77 | 1.00 | 0.89 | 1.12 | 0.63 | 0.48 | 0.83 |
| TLR2 | 1.37 | 1.1 | 1.71 | 1.13 | 1.03 | 1.22 | 0.89 | 0.74 | 1.08 |
| TLR3 | 0.68 | 0.56 | 0.82 | 0.86 | 0.79 | 0.94 | 0.59 | 0.49 | 0.71 |
| TLR4 | 0.88 | 0.73 | 1.07 | 1.04 | 0.95 | 1.15 | 0.7 | 0.53 | 0.92 |
| TNF | 0.88 | 0.72 | 1.07 | 0.91 | 0.83 | 0.99 | 0.71 | 0.58 | 0.87 |
| TP53 | 0.82 | 0.65 | 1.02 | 1.04 | 0.96 | 1.13 | 0.72 | 0.59 | 0.89 |
| TRAF3 | 0.54 | 0.44 | 0.66 | 0.95 | 0.83 | 1.09 | 0.67 | 0.56 | 0.81 |
| TRAF6 | 0.59 | 0.48 | 0.72 | 0.99 | 0.91 | 1.08 | 0.64 | 0.48 | 0.86 |
| TYK2 | 0.69 | 0.56 | 0.86 | 0.90 | 0.79 | 1.02 | 0.74 | 0.61 | 0.9 |
| VDAC3 | 1.25 | 1.04 | 1.52 | 1.59 | 1.11 | 2.29 | 1.45 | 1.2 | 1.75 |
| YWHAB | 1.13 | 0.9 | 1.42 | 1.11 | 0.86 | 1.44 | 1.37 | 1.13 | 1.66 |
| YWHAQ | 1.51 | 1.25 | 1.82 | 1.36 | 0.89 | 2.05 | 1.5 | 1.23 | 1.83 |
| YWHAZ | 1.57 | 1.3 | 1.91 | 1.71 | 0.89 | 3.29 | 1.62 | 1.34 | 1.96 |

Table S6 HBV related genes between breast cancer and normal breast tissues based on RNA seq analysis

| Gene | Fold Change | P value |
| --- | --- | --- |
| BAX | 1.53 | 0.02 |
| CCNE1 | 3.06 | 0.048 |
| E2F1 | 3.49 | 0.04 |
| E2F2 | 3.30 | 0.03 |
| FADD | 1.49 | 0.03 |
| GRB2 | 0.49 | 0.02 |
| IKBKE | 2.16 | 0.03 |
| IRF7 | 2.41 | 0.04 |
| STAT1 | 2.75 | 0.049 |
| AKT3 | 0.51 | 0.04 |
| CASP12 | 0.15 | 0.04 |
| CREB5 | 0.34 | 0.02 |
| JAK1 | 0.66 | 0.03 |
| MAPK11 | 0.68 | 0.04 |
| PIK3R1 | 0.17 | 0.03 |
| TGFBR2 | 0.26 | 0.01 |
| TRAF6 | 0.66 | 0.02 |
| IRAK1 | 1.64 | 0.00 |
| RELA | 0.74 | 0.02 |

Table S7 KEGG enrichment analysis for differentiated expressed genes between breast cancer and normal breast tissues based on RNAseq analysis

| geneSet | description | pValue | Gene |
| --- | --- | --- | --- |
| hsa05161 | Hepatitis B | 3.03E-33 | BAX;CCNE1;E2F1;E2F2;FADD;GRB2;IKBKE;IRF7;STAT1;AKT3;CASP12;CREB5;JAK1;MAPK11;PIK3R1;TGFBR2;TRAF6;IRAK1;RELA |
| hsa05169 | Epstein-Barr virus infection | 2.71E-21 | BAX;CCNE1;E2F1;E2F2;FADD;IKBKE;IRF7;STAT1;AKT3;JAK1;MAPK11;PIK3R1;TRAF6;IRAK1;RELA |
| hsa05160 | Hepatitis C | 8.93E-19 | BAX;E2F1;E2F2;FADD;GRB2;IKBKE;IRF7;STAT1;AKT3;JAK1;PIK3R1;TRAF6;RELA |
| hsa05162 | Measles | 2.52E-17 | BAX;CCNE1;FADD;IKBKE;IRF7;STAT1;AKT3;JAK1;PIK3R1;TRAF6;IRAK1;RELA |
| hsa04620 | Toll-like receptor signaling pathway | 1.14E-16 | FADD;IKBKE;IRF7;STAT1;AKT3;JAK1;MAPK11;PIK3R1;TRAF6;IRAK1;RELA |
| hsa05167 | Kaposi sarcoma-associated herpesvirus infection | 1.46E-15 | BAX;E2F1;E2F2;FADD;IKBKE;IRF7;STAT1;AKT3;JAK1;MAPK11;PIK3R1;RELA |
| hsa05212 | Pancreatic cancer | 4.03E-14 | BAX;E2F1;E2F2;STAT1;AKT3;JAK1;PIK3R1;TGFBR2;RELA |
| hsa05163 | Human cytomegalovirus infection | 4.86E-13 | BAX;E2F1;E2F2;FADD;GRB2;AKT3;CREB5;JAK1;MAPK11;PIK3R1;RELA |
| hsa05165 | Human papillomavirus infection | 8.66E-13 | BAX;CCNE1;E2F1;FADD;GRB2;IKBKE;STAT1;AKT3;CREB5;JAK1;PIK3R1;RELA |
| hsa05220 | Chronic myeloid leukemia | 3.76E-12 | BAX;E2F1;E2F2;GRB2;AKT3;PIK3R1;TGFBR2;RELA |
| hsa04380 | Osteoclast differentiation | 8.25E-12 | GRB2;STAT1;AKT3;JAK1;MAPK11;PIK3R1;TGFBR2;TRAF6;RELA |
| hsa05200 | Pathways in cancer | 8.79E-12 | BAX;CCNE1;E2F1;E2F2;FADD;GRB2;STAT1;AKT3;JAK1;PIK3R1;TGFBR2;TRAF6;RELA |
| hsa05222 | Small cell lung cancer | 1.66E-11 | BAX;CCNE1;E2F1;E2F2;AKT3;PIK3R1;TRAF6;RELA |
| hsa05166 | Human T-cell leukemia virus 1 infection | 1.86E-11 | BAX;CCNE1;E2F1;E2F2;AKT3;CREB5;JAK1;PIK3R1;TGFBR2;RELA |
| hsa05215 | Prostate cancer | 2.81E-11 | CCNE1;E2F1;E2F2;GRB2;AKT3;CREB5;PIK3R1;RELA |
| hsa05142 | Chagas disease | 4.23E-11 | FADD;AKT3;MAPK11;PIK3R1;TGFBR2;TRAF6;IRAK1;RELA |
| hsa05164 | Influenza A | 6.00E-11 | BAX;FADD;IKBKE;IRF7;STAT1;AKT3;JAK1;PIK3R1;RELA |
| hsa05152 | Tuberculosis | 1.06E-10 | BAX;FADD;STAT1;AKT3;JAK1;MAPK11;TRAF6;IRAK1;RELA |
| hsa04621 | NOD-like receptor signaling pathway | 1.30E-10 | FADD;IKBKE;IRF7;STAT1;CASP12;JAK1;MAPK11;TRAF6;RELA |
| hsa04722 | Neurotrophin signaling pathway | 1.48E-10 | BAX;GRB2;AKT3;MAPK11;PIK3R1;TRAF6;IRAK1;RELA |
| hsa05417 | Lipid and atherosclerosis | 5.26E-10 | BAX;IKBKE;IRF7;AKT3;MAPK11;PIK3R1;TRAF6;IRAK1;RELA |
| hsa05226 | Gastric cancer | 7.69E-10 | BAX;CCNE1;E2F1;E2F2;GRB2;AKT3;PIK3R1;TGFBR2 |
| hsa05235 | PD-L1 expression and PD-1 checkpoint pathway in cancer | 8.73E-10 | STAT1;AKT3;JAK1;MAPK11;PIK3R1;TRAF6;RELA |
| hsa04218 | Cellular senescence | 1.31E-09 | CCNE1;E2F1;E2F2;AKT3;MAPK11;PIK3R1;TGFBR2;RELA |
| hsa01522 | Endocrine resistance | 1.50E-09 | BAX;E2F1;E2F2;GRB2;AKT3;MAPK11;PIK3R1 |
| hsa05168 | Herpes simplex virus 1 infection | 1.59E-09 | BAX;FADD;IKBKE;IRF7;STAT1;AKT3;JAK1;PIK3R1;TRAF6;IRAK1;RELA |
| hsa04933 | AGE-RAGE signaling pathway in diabetic complications | 2.00E-09 | BAX;STAT1;AKT3;MAPK11;PIK3R1;TGFBR2;RELA |
| hsa05145 | Toxoplasmosis | 4.18E-09 | STAT1;AKT3;JAK1;MAPK11;TRAF6;IRAK1;RELA |
| hsa05203 | Viral carcinogenesis | 7.38E-09 | BAX;CCNE1;GRB2;IRF7;CREB5;JAK1;PIK3R1;RELA |
| hsa04917 | Prolactin signaling pathway | 1.02E-08 | GRB2;STAT1;AKT3;MAPK11;PIK3R1;RELA |
| hsa04622 | RIG-I-like receptor signaling pathway | 1.11E-08 | FADD;IKBKE;IRF7;MAPK11;TRAF6;RELA |
| hsa04926 | Relaxin signaling pathway | 1.14E-08 | GRB2;AKT3;CREB5;MAPK11;PIK3R1;TGFBR2;RELA |
| hsa05223 | Non-small cell lung cancer | 1.21E-08 | BAX;E2F1;E2F2;GRB2;AKT3;PIK3R1 |
| hsa05170 | Human immunodeficiency virus 1 infection | 1.38E-08 | BAX;FADD;AKT3;MAPK11;PIK3R1;TRAF6;IRAK1;RELA |
| hsa05214 | Glioma | 1.55E-08 | BAX;E2F1;E2F2;GRB2;AKT3;PIK3R1 |
| hsa05140 | Leishmaniasis | 1.68E-08 | STAT1;JAK1;MAPK11;TRAF6;IRAK1;RELA |
| hsa04936 | Alcoholic liver disease | 2.23E-08 | FADD;IKBKE;AKT3;MAPK11;TRAF6;IRAK1;RELA |
| hsa05171 | Coronavirus disease | 2.72E-08 | IKBKE;STAT1;JAK1;MAPK11;PIK3R1;TRAF6;IRAK1;RELA |
| hsa05225 | Hepatocellular carcinoma | 6.10E-08 | BAX;E2F1;E2F2;GRB2;AKT3;PIK3R1;TGFBR2 |
| hsa04625 | C-type lectin receptor signaling pathway | 9.99E-08 | IKBKE;STAT1;AKT3;MAPK11;PIK3R1;RELA |
| hsa04668 | TNF signaling pathway | 1.94E-07 | FADD;AKT3;CREB5;MAPK11;PIK3R1;RELA |
| hsa05207 | Chemical carcinogenesis | 2.24E-07 | E2F1;GRB2;IKBKE;AKT3;CREB5;PIK3R1;RELA |
| hsa04935 | Growth hormone synthesis, secretion and action | 2.64E-07 | GRB2;STAT1;AKT3;CREB5;MAPK11;PIK3R1 |
| hsa04210 | Apoptosis | 5.55E-07 | BAX;FADD;AKT3;CASP12;PIK3R1;RELA |
| hsa05135 | Yersinia infection | 5.55E-07 | AKT3;MAPK11;PIK3R1;TRAF6;IRAK1;RELA |
| hsa05218 | Melanoma | 6.02E-07 | BAX;E2F1;E2F2;AKT3;PIK3R1 |
| hsa05224 | Breast cancer | 8.11E-07 | BAX;E2F1;E2F2;GRB2;AKT3;PIK3R1 |
| hsa01521 | EGFR tyrosine kinase inhibitor resistance | 9.00E-07 | BAX;GRB2;AKT3;JAK1;PIK3R1 |
| hsa05132 | Salmonella infection | 1.04E-06 | BAX;FADD;AKT3;MAPK11;TRAF6;IRAK1;RELA |
| hsa05210 | Colorectal cancer | 1.47E-06 | BAX;GRB2;AKT3;PIK3R1;TGFBR2 |
| hsa04211 | Longevity regulating pathway | 1.65E-06 | BAX;AKT3;CREB5;PIK3R1;RELA |
| hsa04657 | IL-17 signaling pathway | 2.29E-06 | FADD;IKBKE;MAPK11;TRAF6;RELA |
| hsa04010 | MAPK signaling pathway | 3.75E-06 | GRB2;AKT3;MAPK11;TGFBR2;TRAF6;IRAK1;RELA |
| hsa05130 | Pathogenic Escherichia coli infection | 4.19E-06 | BAX;FADD;MAPK11;TRAF6;IRAK1;RELA |
| hsa04659 | Th17 cell differentiation | 4.54E-06 | STAT1;JAK1;MAPK11;TGFBR2;RELA |
| hsa04071 | Sphingolipid signaling pathway | 7.63E-06 | BAX;AKT3;MAPK11;PIK3R1;RELA |
| hsa04660 | T cell receptor signaling pathway | 7.95E-06 | GRB2;AKT3;MAPK11;PIK3R1;RELA |
| hsa05213 | Endometrial cancer | 9.69E-06 | BAX;GRB2;AKT3;PIK3R1 |
| hsa04068 | FoxO signaling pathway | 1.17E-05 | GRB2;AKT3;MAPK11;PIK3R1;TGFBR2 |
| hsa04151 | PI3K-Akt signaling pathway | 1.19E-05 | CCNE1;GRB2;AKT3;CREB5;JAK1;PIK3R1;RELA |
| hsa04664 | Fc epsilon RI signaling pathway | 1.53E-05 | GRB2;AKT3;MAPK11;PIK3R1 |
| hsa05131 | Shigellosis | 1.55E-05 | BAX;AKT3;MAPK11;PIK3R1;TRAF6;RELA |
| hsa04550 | Signaling pathways regulating pluripotency of stem cells | 1.68E-05 | GRB2;AKT3;JAK1;MAPK11;PIK3R1 |
| hsa05221 | Acute myeloid leukemia | 1.73E-05 | GRB2;AKT3;PIK3R1;RELA |
| hsa01524 | Platinum drug resistance | 2.17E-05 | BAX;FADD;AKT3;PIK3R1 |
| hsa04623 | Cytosolic DNA-sensing pathway | 2.43E-05 | FADD;IKBKE;IRF7;RELA |
| hsa04932 | Non-alcoholic fatty liver disease | 2.49E-05 | BAX;AKT3;MAPK11;PIK3R1;RELA |
| hsa05133 | Pertussis | 2.85E-05 | MAPK11;TRAF6;IRAK1;RELA |
| hsa04630 | JAK-STAT signaling pathway | 3.59E-05 | GRB2;STAT1;AKT3;JAK1;PIK3R1 |
| hsa04662 | B cell receptor signaling pathway | 4.04E-05 | GRB2;AKT3;PIK3R1;RELA |
| hsa04658 | Th1 and Th2 cell differentiation | 6.05E-05 | STAT1;JAK1;MAPK11;RELA |
| hsa04062 | Chemokine signaling pathway | 6.70E-05 | GRB2;STAT1;AKT3;PIK3R1;RELA |
| hsa05206 | MicroRNAs in cancer | 6.87E-05 | CCNE1;E2F1;E2F2;GRB2;PIK3R1 |
| hsa05415 | Diabetic cardiomyopathy | 9.21E-05 | AKT3;MAPK11;PIK3R1;TGFBR2;RELA |
| hsa04931 | Insulin resistance | 1.13E-04 | AKT3;CREB5;PIK3R1;RELA |
| hsa05208 | Chemical carcinogenesis | 1.41E-04 | GRB2;AKT3;MAPK11;PIK3R1;RELA |
| hsa04915 | Estrogen signaling pathway | 2.61E-04 | GRB2;AKT3;CREB5;PIK3R1 |
| hsa05418 | Fluid shear stress and atherosclerosis | 2.76E-04 | AKT3;MAPK11;PIK3R1;RELA |
| hsa04370 | VEGF signaling pathway | 3.27E-04 | AKT3;MAPK11;PIK3R1 |
| hsa05020 | Prion disease | 3.68E-04 | BAX;CASP12;CREB5;MAPK11;PIK3R1 |
| hsa04217 | Necroptosis | 4.22E-04 | BAX;FADD;STAT1;JAK1 |
| hsa04934 | Cushing syndrome | 4.44E-04 | CCNE1;E2F1;E2F2;CREB5 |
| hsa05211 | Renal cell carcinoma | 5.50E-04 | GRB2;AKT3;PIK3R1 |
| hsa04613 | Neutrophil extracellular trap formation | 6.89E-04 | AKT3;MAPK11;PIK3R1;RELA |
| hsa04012 | ErbB signaling pathway | 0.001056387 | GRB2;AKT3;PIK3R1 |
| hsa05205 | Proteoglycans in cancer | 0.001230941 | GRB2;AKT3;MAPK11;PIK3R1 |
| hsa04914 | Progesterone-mediated oocyte maturation | 0.001328781 | AKT3;MAPK11;PIK3R1 |
| hsa05231 | Choline metabolism in cancer | 0.001502584 | GRB2;AKT3;PIK3R1 |
| hsa05010 | Alzheimer disease | 0.001695454 | FADD;AKT3;CASP12;PIK3R1;RELA |
| hsa04064 | NF-kappa B signaling pathway | 0.001739165 | TRAF6;IRAK1;RELA |
| hsa04024 | cAMP signaling pathway | 0.001774868 | AKT3;CREB5;PIK3R1;RELA |
| hsa04066 | HIF-1 signaling pathway | 0.001997932 | AKT3;PIK3R1;RELA |
| hsa04014 | Ras signaling pathway | 0.002085223 | GRB2;AKT3;PIK3R1;RELA |
| hsa04725 | Cholinergic synapse | 0.002398873 | AKT3;CREB5;PIK3R1 |
| hsa04215 | Apoptosis | 0.002489496 | BAX;FADD |
| hsa04152 | AMPK signaling pathway | 0.002914547 | AKT3;CREB5;PIK3R1 |
| hsa04611 | Platelet activation | 0.002914547 | AKT3;MAPK11;PIK3R1 |
| hsa04919 | Thyroid hormone signaling pathway | 0.002914547 | STAT1;AKT3;PIK3R1 |
| hsa04728 | Dopaminergic synapse | 0.003728355 | AKT3;CREB5;MAPK11 |
| hsa04910 | Insulin signaling pathway | 0.004055471 | GRB2;AKT3;PIK3R1 |
| hsa05219 | Bladder cancer | 0.004327093 | E2F1;E2F2 |
| hsa05022 | Pathways of neurodegeneration | 0.004358929 | BAX;FADD;CASP12;MAPK11;RELA |
| hsa04072 | Phospholipase D signaling pathway | 0.004854392 | GRB2;AKT3;PIK3R1 |
| hsa04973 | Carbohydrate digestion and absorption | 0.005422339 | AKT3;PIK3R1 |
| hsa04150 | mTOR signaling pathway | 0.00563964 | GRB2;AKT3;PIK3R1 |
| hsa04261 | Adrenergic signaling in cardiomyocytes | 0.005742872 | AKT3;CREB5;MAPK11 |
| hsa04110 | Cell cycle | 0.005952758 | CCNE1;E2F1;E2F2 |
| hsa05030 | Cocaine addiction | 0.00613441 | CREB5;RELA |
| hsa04140 | Autophagy | 0.006838478 | AKT3;PIK3R1;TRAF6 |
| hsa04923 | Regulation of lipolysis in adipocytes | 0.008511355 | AKT3;PIK3R1 |
| hsa04213 | Longevity regulating pathway | 0.009382057 | AKT3;PIK3R1 |
| hsa05202 | Transcriptional misregulation in cancer | 0.009808967 | BAX;TGFBR2;RELA |
| hsa04929 | GnRH secretion | 0.010291039 | AKT3;PIK3R1 |
| hsa05321 | Inflammatory bowel disease | 0.010602445 | STAT1;RELA |
| hsa04920 | Adipocytokine signaling pathway | 0.011889603 | AKT3;RELA |
| hsa04510 | Focal adhesion | 0.012097292 | GRB2;AKT3;PIK3R1 |
| hsa05120 | Epithelial cell signaling in Helicobacter pylori infection | 0.012221676 | MAPK11;RELA |
| hsa05230 | Central carbon metabolism in cancer | 0.012221676 | AKT3;PIK3R1 |
| hsa04015 | Rap1 signaling pathway | 0.013092354 | AKT3;MAPK11;PIK3R1 |
| hsa04115 | p53 signaling pathway | 0.013590519 | BAX;CCNE1 |
| hsa04714 | Thermogenesis | 0.01733746 | GRB2;CREB5;MAPK11 |
| hsa04912 | GnRH signaling pathway | 0.020106741 | GRB2;MAPK11 |
| hsa04666 | Fc gamma R-mediated phagocytosis | 0.021369562 | AKT3;PIK3R1 |
| hsa04750 | Inflammatory mediator regulation of TRP channels | 0.022229583 | MAPK11;PIK3R1 |
| hsa04137 | Mitophagy | 0.024442075 | E2F1;RELA |
| hsa05146 | Amoebiasis | 0.024442075 | PIK3R1;RELA |
| hsa04922 | Glucagon signaling pathway | 0.02721226 | AKT3;CREB5 |
| hsa04670 | Leukocyte transendothelial migration | 0.029127109 | MAPK11;PIK3R1 |
| hsa04114 | Oocyte meiosis | 0.034144405 | CCNE1;MAPK11 |
| hsa04650 | Natural killer cell mediated cytotoxicity | 0.036240558 | GRB2;PIK3R1 |
| hsa05017 | Spinocerebellar ataxia | 0.044532637 | AKT3;PIK3R1 |

1. Chen DT, Nasir A, Culhane A, et al. Proliferative genes dominate malignancy-risk gene signature in histologically-normal breast tissue. Breast Cancer Res Treat, 2010. 119(2): 335-46.

2. Cedro-Tanda A, Ríos-Romero M, Romero-Córdoba S. A lncRNA landscape in breast cancer reveals a potential role for AC009283.1 in proliferation and apoptosis in HER2-enriched subtype. 2020. 10(1): 13146.

3. Haakensen VD, Biong M, Lingjærde OC, et al. Expression levels of uridine 5'-diphospho-glucuronosyltransferase genes in breast tissue from healthy women are associated with mammographic density. Breast Cancer Res, 2010. 12(4): R65.

4. Kretschmer C, Sterner-Kock A, Siedentopf F, et al. Identification of early molecular markers for breast cancer. Mol Cancer, 2011. 10(1): 15.

5. Colak D, Nofal A, Albakheet A, et al. Age-specific gene expression signatures for breast tumors and cross-species conserved potential cancer progression markers in young women. PLoS One, 2013. 8(5): e63204.

6. Lian ZQ, Wang Q, Li WP, et al. Screening of significantly hypermethylated genes in breast cancer using microarray-based methylated-CpG island recovery assay and identification of their expression levels. Int J Oncol, 2012. 41(2): 629-38.

7. Maire V, Némati F, Richardson M, et al. Polo-like kinase 1: a potential therapeutic option in combination with conventional chemotherapy for the management of patients with triple-negative breast cancer. Cancer Res, 2013. 73(2): 813-23.

8. Merdad A, Karim S, Schulten HJ, et al. Expression of matrix metalloproteinases (MMPs) in primary human breast cancer: MMP-9 as a potential biomarker for cancer invasion and metastasis. Anticancer Res, 2014. 34(3): 1355-66.

9. Pau Ni IB, Zakaria Z, Muhammad R, et al. Gene expression patterns distinguish breast carcinomas from normal breast tissues: the Malaysian context. Pathol Res Pract, 2010. 206(4): 223-8.

10. Uva P, Aurisicchio L, Watters J, et al. Comparative expression pathway analysis of human and canine mammary tumors. BMC Genomics, 2009. 10: 135.

11. Román-Pérez E, Casbas-Hernández P, Pirone JR, et al. Gene expression in extratumoral microenvironment predicts clinical outcome in breast cancer patients. Breast Cancer Res, 2012. 14(2): R51.

12. Clarke C, Madden SF, Doolan P, et al. Correlating transcriptional networks to breast cancer survival: a large-scale coexpression analysis. Carcinogenesis, 2013. 34(10): 2300-8.

13. Pedersen IS, Thomassen M, Tan Q, et al. Differential effect of surgical manipulation on gene expression in normal breast tissue and breast tumor tissue. Mol Med, 2018. 24(1): 57.

14. Chang JW, Kuo WH, Lin CM, et al. Wild-type p53 upregulates an early onset breast cancer-associated gene GAS7 to suppress metastasis via GAS7-CYFIP1-mediated signaling pathway. 2018. 37(30): 4137-4150.

15. Loi S, Haibe-Kains B, Desmedt C, et al. Predicting prognosis using molecular profiling in estrogen receptor-positive breast cancer treated with tamoxifen. BMC Genomics, 2008. 9: 239.

16. Pawitan Y, Bjöhle J, Amler L, et al. Gene expression profiling spares early breast cancer patients from adjuvant therapy: derived and validated in two population-based cohorts. Breast Cancer Res, 2005. 7(6): R953-64.

17. Wang Y, Klijn JG, Zhang Y, et al. Gene-expression profiles to predict distant metastasis of lymph-node-negative primary breast cancer. Lancet, 2005. 365(9460): 671-9.

18. Minn AJ, Gupta GP, Siegel PM, et al. Genes that mediate breast cancer metastasis to lung. Nature, 2005. 436(7050): 518-24.

19. Bild AH, Yao G, Chang JT, et al. Oncogenic pathway signatures in human cancers as a guide to targeted therapies. Nature, 2006. 439(7074): 353-7.

20. Miller LD, Smeds J, George J, et al. An expression signature for p53 status in human breast cancer predicts mutation status, transcriptional effects, and patient survival. Proc Natl Acad Sci U S A, 2005. 102(38): 13550-5.

21. Ivshina AV, George J, Senko O, et al. Genetic reclassification of histologic grade delineates new clinical subtypes of breast cancer. Cancer Res, 2006. 66(21): 10292-301.

22. Minn AJ, Gupta GP, Padua D, et al. Lung metastasis genes couple breast tumor size and metastatic spread. Proc Natl Acad Sci U S A, 2007. 104(16): 6740-5.

23. Loi S, Haibe-Kains B, Desmedt C, et al. Definition of clinically distinct molecular subtypes in estrogen receptor-positive breast carcinomas through genomic grade. J Clin Oncol, 2007. 25(10): 1239-46.

24. Zhou Y, Yau C, Gray JW, et al. Enhanced NF kappa B and AP-1 transcriptional activity associated with antiestrogen resistant breast cancer. BMC Cancer, 2007. 7: 59.

25. Desmedt C, Piette F, Loi S, et al. Strong time dependence of the 76-gene prognostic signature for node-negative breast cancer patients in the TRANSBIG multicenter independent validation series. Clin Cancer Res, 2007. 13(11): 3207-14.

26. Anders CK, Acharya CR, Hsu DS, et al. Age-specific differences in oncogenic pathway deregulation seen in human breast tumors. PLoS One, 2008. 3(1): e1373.

27. Chanrion M, Negre V, Fontaine H, et al. A gene expression signature that can predict the recurrence of tamoxifen-treated primary breast cancer. Clin Cancer Res, 2008. 14(6): 1744-52.

28. Schmidt M, Böhm D, von Törne C, et al. The humoral immune system has a key prognostic impact in node-negative breast cancer. Cancer Res, 2008. 68(13): 5405-13.

29. Zhang Y, Sieuwerts AM, McGreevy M, et al. The 76-gene signature defines high-risk patients that benefit from adjuvant tamoxifen therapy. Breast Cancer Res Treat, 2009. 116(2): 303-9.

30. Desmedt C, Di Leo A, de Azambuja E, et al. Multifactorial approach to predicting resistance to anthracyclines. J Clin Oncol, 2011. 29(12): 1578-86.

31. Symmans WF, Hatzis C, Sotiriou C, et al. Genomic index of sensitivity to endocrine therapy for breast cancer. J Clin Oncol, 2010. 28(27): 4111-9.

32. Sircoulomb F, Bekhouche I, Finetti P, et al. Genome profiling of ERBB2-amplified breast cancers. BMC Cancer, 2010. 10: 539.

33. Li Y, Zou L, Li Q, et al. Amplification of LAPTM4B and YWHAZ contributes to chemotherapy resistance and recurrence of breast cancer. Nat Med, 2010. 16(2): 214-8.

34. Kao KJ, Chang KM, Hsu HC, et al. Correlation of microarray-based breast cancer molecular subtypes and clinical outcomes: implications for treatment optimization. BMC Cancer, 2011. 11: 143.

35. Dedeurwaerder S, Desmedt C, Calonne E, et al. DNA methylation profiling reveals a predominant immune component in breast cancers. EMBO Mol Med, 2011. 3(12): 726-41.

36. Sabatier R, Finetti P, Cervera N, et al. A gene expression signature identifies two prognostic subgroups of basal breast cancer. Breast Cancer Res Treat, 2011. 126(2): 407-20.

37. Buffa FM, Camps C, Winchester L, et al. microRNA-associated progression pathways and potential therapeutic targets identified by integrated mRNA and microRNA expression profiling in breast cancer. Cancer Res, 2011. 71(17): 5635-45.

38. Hatzis C, Pusztai L, Valero V, et al. A genomic predictor of response and survival following taxane-anthracycline chemotherapy for invasive breast cancer. Jama, 2011. 305(18): 1873-81.

39. Nagalla S, Chou JW, Willingham MC, et al. Interactions between immunity, proliferation and molecular subtype in breast cancer prognosis. Genome Biol, 2013. 14(4): R34.

40. Huang CC, Tu SH, Lien HH, et al. Concurrent gene signatures for han chinese breast cancers. PLoS One, 2013. 8(10): e76421.

41. Jézéquel P, Loussouarn D, Guérin-Charbonnel C, et al. Gene-expression molecular subtyping of triple-negative breast cancer tumours: importance of immune response. Breast Cancer Res, 2015. 17: 43.

42. Grinchuk OV, Motakis E, Yenamandra SP, et al. Sense-antisense gene-pairs in breast cancer and associated pathological pathways. Oncotarget, 2015. 6(39): 42197-221.

43. Chin K, DeVries S, Fridlyand J, et al. Genomic and transcriptional aberrations linked to breast cancer pathophysiologies. Cancer Cell, 2006. 10(6): 529-41.

44. Metzger-Filho O, Michiels S, Bertucci F, et al. Genomic grade adds prognostic value in invasive lobular carcinoma. Ann Oncol, 2013. 24(2): 377-384.

45. Heimes AS, Härtner F, Almstedt K, et al. Prognostic Significance of Interferon-γ and Its Signaling Pathway in Early Breast Cancer Depends on the Molecular Subtypes. 2020. 21(19).

46. Jönsson G, Staaf J, Vallon-Christersson J, et al. The retinoblastoma gene undergoes rearrangements in BRCA1-deficient basal-like breast cancer. Cancer Res, 2012. 72(16): 4028-36.

47. Harrell JC, Prat A, Parker JS, et al. Genomic analysis identifies unique signatures predictive of brain, lung, and liver relapse. Breast Cancer Res Treat, 2012. 132(2): 523-35.

48. Callari M, Musella V, Di Buduo E, et al. Subtype-dependent prognostic relevance of an interferon-induced pathway metagene in node-negative breast cancer. Mol Oncol, 2014. 8(7): 1278-89.

49. Wang DY, Done SJ, Mc Cready DR, et al. Validation of the prognostic gene portfolio, ClinicoMolecular Triad Classification, using an independent prospective breast cancer cohort and external patient populations. Breast Cancer Res, 2014. 16(4): R71.

50. Jonsdottir K, Assmus J, Slewa A, et al. Prognostic value of gene signatures and proliferation in lymph-node-negative breast cancer. PLoS One, 2014. 9(3): e90642.

51. Azim HA, Jr., Brohée S, Peccatori FA, et al. Biology of breast cancer during pregnancy using genomic profiling. Endocr Relat Cancer, 2014. 21(4): 545-54.

52. Prabhakaran S, Rizk VT, Ma Z, et al. Evaluation of invasive breast cancer samples using a 12-chemokine gene expression score: correlation with clinical outcomes. Breast Cancer Res, 2017. 19(1): 71.

53. Kensler KH, Sankar VN, Wang J, et al. PAM50 Molecular Intrinsic Subtypes in the Nurses' Health Study Cohorts. 2019. 28(4): 798-806.

54. Kim SK, Ahn SG, Mun JY, et al. Genomic Signature of the Standardized Uptake Value in (18)F-Fluorodeoxyglucose Positron Emission Tomography in Breast Cancer. Cancers (Basel), 2020. 12(2).

55. Chen YJ, Huang CS, Phan NN, et al. Molecular subtyping of breast cancer intrinsic taxonomy with oligonucleotide microarray and NanoString nCounter. 2021. 41(8).

56. Jönsson G, Staaf J, Vallon-Christersson J, et al. Genomic subtypes of breast cancer identified by array-comparative genomic hybridization display distinct molecular and clinical characteristics. Breast Cancer Res, 2010. 12(3): R42.

57. Esserman LJ, Berry DA, Cheang MC, et al. Chemotherapy response and recurrence-free survival in neoadjuvant breast cancer depends on biomarker profiles: results from the I-SPY 1 TRIAL (CALGB 150007/150012; ACRIN 6657). Breast Cancer Res Treat, 2012. 132(3): 1049-62.

58. Prat A, Parker JS, Karginova O, et al. Phenotypic and molecular characterization of the claudin-low intrinsic subtype of breast cancer. Breast Cancer Res, 2010. 12(5): R68.

59. Enerly E, Steinfeld I, Kleivi K, et al. miRNA-mRNA integrated analysis reveals roles for miRNAs in primary breast tumors. PLoS One, 2011. 6(2): e16915.
